# Multimodal Inhibition of *Pectobacterium brasiliense* Virulence by the Citrus Flavanone Naringenin

**DOI:** 10.1021/acs.jafc.5c04312

**Published:** 2025-08-06

**Authors:** Manoj Pun, Ortal Galsurker, Netaly Khazanov, Amy Charkowski, Shani Yelin, Zohar Kerem, Michal Weitman, Hanoch Senderowitz, Iris Yedidia

**Affiliations:** † The Institute of Plant Sciences, Volcani Center, 42718 Agricultural Research Organization (ARO), 7528809 Rishon Lezion, Israel; ‡ The Robert H. Smith Faculty of Agriculture, Food and Environment, The Hebrew University of Jerusalem, 76100 Rehovot, Israel; § Department of Chemistry, Bar-Ilan University, 52900 Ramat Gan, Israel; ∥ Department of Agricultural Biology, Colorado State University, Fort Collins, Colorado 80521, United States

**Keywords:** *Pectobacterium brasiliense*, naringenin, quorum sensing inhibitor, ROS, molecular docking, molecular dynamics

## Abstract

Naringenin, a flavanone from citrus, was studied for its ability to reduce virulence in *Pectobacterium*, a phytopathogen causing soft rot disease in crop plants. Naringenin downregulated quorum sensing (QS) and suppressed critical virulence determinants in *Pectobacterium brasiliense* Pb1692, including plant cell wall-degrading enzymes, bacterial motility, and biofilm formation, consequently reducing disease symptoms in two host plants. Molecular docking simulations revealed a plausible binding mode for naringenin within the QS protein ExpI, which were maintained during microsecond-long Molecular Dynamics simulations. These simulations provided atomic-scale insight into specific interactions and estimated binding free energies, supporting naringenin’s QS inhibition mode of action. In contrast, *S*-adenosyl methionine, the natural ligand of ExpI, was unable to maintain a stable binding mode in the ExpI site during simulations. Beyond QS disruption, naringenin induced reactive oxygen species accumulation and compromised DNA repair, indicating a multimodal mechanism of action. Despite these promising findings, naringenin’s limited aqueous solubility challenges practical applications.

## Graphical abstract

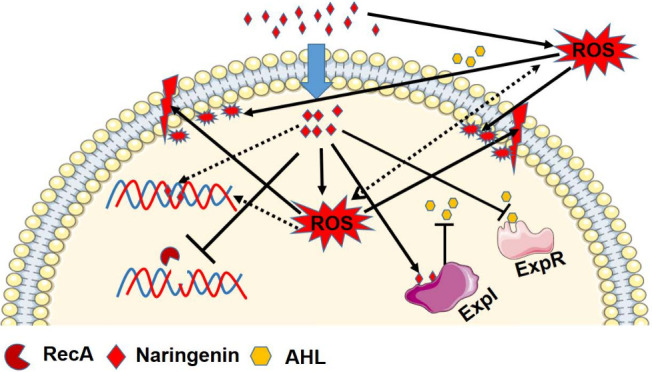

## Introduction

Bacterial soft rot disease significantly impacts agricultural crop systems, particularly affecting potato, vegetables, and ornamental plants while having minimal influence on natural ecosystems. This devastating disease disrupts agricultural production systems, resulting in losses of up to 30% of crops across various stages of the supply chain including planting, growing, transportation, and storage.^1^ Soft rot pectobacteria (SRP), members of the family Pectobacteriaceae, predominantly cause soft rot symptoms. These pathogens primarily target plant storage organs, including tubers, rhizomes, and bulbs, where symptoms are most readily detected.^2,3^ A novel approach to control infections caused by pathogenic bacteria in both plant and animal hosts is targeting the bacterial virulence regulatory mechanism known as the quorum sensing (QS) system.^4–7^ Unlike antibiotics, which impose strong selective pressure on bacterial communities, quorum sensing inhibitors (QSI) do not kill bacteria but rather impair their infection capability by disrupting bacterial communication systems, thereby reducing the likelihood of resistance development.^8–10^ In *Pectobacterium*, the primary cause of soft rot disease, quorum sensing (QS) serves as a key mechanism regulating virulence through the production of plant cell wall degrading enzymes (PCWDEs), which are predominantly responsible for disease symptom development. This regulatory system operates based on population density, controlling gene expression through diffusible signaling molecules.^11–13^ *N*-Acylhomoserine lactones (AHLs) particularly 3-oxo-C6-HSL and 3-oxo-C8-HSL, function as the primary signaling compounds in Pectobacteria.^15^ These molecules are synthesized by the ExpI protein (AHL synthase) and detected by response regulators ExpR1 and ExpR2 proteins, which negatively regulate PCWDEs production.^14,15^

Throughout their evolutionary history, plants have developed a diverse array of secondary metabolites as part of a sophisticated defense network against pathogenic microorganisms. These defensive compounds include phenolic acids, quinones, flavonoids, terpenoids, and alkaloids.^16^ While many plant-derived substances exhibit mild antimicrobial activity, certain compounds show particular promise in inhibiting bacterial virulence. Specifically, several natural compounds effectively disrupt quorum sensing, presenting a potential strategy for managing bacterial diseases such as bacterial soft rot.^16,17^ One notable example is naringenin, a flavonoid abundant in citrus fruits, including grapefruit, orange, and tangerines. This compound plays a significant role in plant defense against pathogens,^18^ and demonstrates antimicrobial properties, particularly against the pear and apple tree pathogen *Erwinia amylovora*.^18–20^ Additionally, naringenin, a natural constituent of the human diet, has demonstrated therapeutic potential in mammalian models for various inflammation-related diseases, including sepsis, hepatitis, fibrosis, and cancer, making it a promising candidate for further investigation.^21–23^

Correspondingly, in this work, we evaluated naringenin’s antimicrobial properties against *Pectobacterium brasiliense* (Pb1692), focusing on its impact on quorum sensing and virulence. We assessed its effects on bacterial growth, motility, biofilm formation, plant cell-wall degrading enzyme (PCWDE) secretion, and infection in potato and calla lilies, two soft rot susceptible crops. To provide atomic-scale mechanistic insights into the mechanism of action of naringenin, we used molecular docking and molecular dynamics simulations to study, for the first time, the ability of naringenin to compete with the natural precursors of AHL, *S*-adenosyl-l-methionine (SAM) and acyl–acyl carrier protein (ACCP) in the active binding sites of ExpI, by this disrupting AHL production. Our findings suggest that naringenin not only disrupts quorum sensing-dependent virulence but also enhances reactive oxygen species (ROS) formation, and triggers an oxidative stress response in the pathogen, supporting a multimodal mode of action.

## Methods

### Bacterial Strains, Growth Media, and Chemicals

The *Escherichia coli* and *P. brasiliense* strains used in this study are listed in Supporting Information (SI), Table S1. All strains were grown in a Luria–Bertani (LB) medium (Difco Laboratories, MI, USA). *E. coli* strains *pSB401* and *DH5α* were cultivated at 37 °C, while Pb1692 and CV026 strains were grown at 28 °C with continuous shaking (150 rpm) in a TU- incubator (MRC, Holon, Israel). For the infection assay, Murashige and Skoog (MS) minimal medium plates (Duchefa, Haarlem, The Netherlands) were used.

Solvents and chemicals were purchased from Sigma-Aldrich. Naringenin was dissolved in DMSO to prepare stock solution of either 200 or 300 mM to maintain low DMSO concentration in LB medium. Experimental concentrations were prepared by gradually diluting naringenin in DMSO, with a final concentration of 25 mM at 10% DMSO. Where required, the following antibiotics were added: kanamycin (10 μg/mL), tetracycline (10 μg/mL), and ampicillin (100 μg/mL).

### Growth Curves

The growth curves of Pb1692 were analyzed in a 96-well microtiter plate under increasing naringenin concentrations. A 300 mM working stock of naringenin was prepared in DMSO. Bacterial cultures were grown for 16 h at 28 °C with continuous shaking (150 rpm), washed once by centrifuging (3 min, 12,000 rpm), and resuspended in fresh LB to an OD_600_ of 0.1 (10^8^ CFU/ml).

Experimental wells received 190 μL LB with naringenin (0.5–3.0 mM), while controls contained LB without naringenin or with DMSO (0.8% in LB). A 10 μL bacterial suspension (10^8^ CFU/mL) was added to each well, resulting in a final concentration of 5 × 10^6^ CFU/mL in 200 μL total volume. Plates were incubated at 28 °C in a Tecan Spark multimode microplate reader (Tecan Trading AG, Switzerland), with OD_600_ recorded hourly for 24 h. The experiment was performed in triplicate with four replicates per treatment.

### Luminescence Assay

To quantify AHL production, *E. coli* strain pSB401 was used as a reporter, generating luminescence in response to 3-oxo-C6-HSL.^24^ Single colonies of Pb1692 and pSB401 were grown overnight in LB at 28 and 37 °C respectively, under continuous shaking (150 rpm). Tetracycline (10 μg/mL) was added to LB for the reporter pSB401 strain. Pb1692 culture was washed, adjusted to 5 × 10^6^ CFU/mL, and incubated with 0.5–2.0 mM naringenin at 28 °C for 8 h under continuous shaking (150 rpm). Meanwhile, pSB401 was diluted 1:5 in fresh LB containing tetracycline (10 μg/mL) and incubated at 37 °C for 8 h. After incubation, Pb1692 cultures were centrifuged (5 min, 14,000 rpm, RT) and 20 μL of each supernatant was mixed with 180 μL of pSB401 (5 × 10^6^ CFU/mL) in a 96-well microtiter plate. A positive control contained pSB401 and 50 nM synthetic eAHL (Sigma, St. Louis, MO, USA). The plate was incubated for 18 h at 37 °C in a Tecan Spark multimode microplate reader (Tecan Trading AG, Switzerland), with bioluminescence and OD_600_ measured every 30 min. Bioluminescence was normalized by dividing relative light units (RLU) by OD_600._ ^25^ The assay was performed twice with 8 replicated per treatment.

### AHL Extraction and Quantification

#### AHL Extraction

AHL extraction was performed as described previously, with minor modifications.^26^ Pb1692 (5 × 10^6^ CFU/mL) was cultured in LB with 0.5, 1, and 2 mM of naringenin for 24 h at 28 °C. After incubation, the cultures were centrifuged (8000*g*, 15 min, 4 °C) and AHLs were extracted by adding equal volume of ethyl acetate with 0.1% formic acid (v/v). The clear upper phase was collected and dried using a SpeedVac concentrator (Savant SPD 111 V, Thermo Scientific, MA, United States), resuspended in acetonitrile (Alfa Aesar, United States) and analyzed by LC-MS/MS after diluting into HPLC water (1:5 ratio).

UHPLC was performed on an Agilent 6545 QTOF mass spectrometer with an electrospray ionization (ESI) source. A ZORBAX RRHD Eclipse Plus C18 column was used with a water-MeCN gradient (5–95% MeCN over 10 min) at a flow rate of 0.5 mL/min. The mass spectral parameters were optimized for 3-oxo-C6-HSL and 3-oxo-C8-HSL under identical condition.

#### AHL Detection Using the Reporter Strain *Chromobacterium violaceum*

Qualitative detection of AHL was performed using the reporter strain *C. violaceum* CV026 that produces violacein purple pigment in the presence of AHL compounds with *N*-acyl C4–C8 side chains.^27^ A disc-diffusion assay was used to detect inhibition of AHL production by naringenin, using a previously described procedure.^28,29^ Pb1692 and CV026 were grown for 16 h at 28 °C and centrifuged (14000 rpm, 5 min), and the supernatant was discarded. Both strains CV026 and Pb1692 were resuspended in fresh LB at a concentration of 5 × 10^6^ CFU/mL. A smaller circular ring was made with a Pb1692 suspension, and a surrounding, bigger ring was made with CV026; a paper disc was mounted in the center of the inner ring. Then 30 μL of each concentration of naringenin was gently pipetted onto the paper disc and dried for 30 min in a laminar flow hood. The plates were incubated at 28 °C for 16 h, and the intensity of the purple pigment produced by the reporter strain was assessed.

Finally, to confirm the direct interference of naringenin with ExpI, the AHL synthase of Pb1692 was introduced to a DH5α strain (DH5α /pGEM *expI*), lacking any components of the QS machinery. DH5α /pGEM *expI* and DH5α were grown overnight (ON) at 37 °C with continuous shaking (200 rpm) and used under the above protocol for the disc diffusion assay with CV026 as a reporter strain.

#### Biofilm Formation, Swimming, and Swarming Motility

Biofilm formation was assessed using crystal violet (CV) staining in a 96-well microtiter plate.^30,31^ Pb1692 (5 × 10^6^ CFU) was grown in 2YT (Yeast tryptone; tryptone 17 g/L, NaCl 5 g/L, and yeast broth extract 10 g/L, pH 7.0) with 0.5–2 mM naringenin or control (water or 0.8% DMSO) for 72 h at RT without shaking. Biofilms were stained with Crystal Violet (0.1%), washed, and dissolved in 30% acetic acid before OD_550_ measurement in a Tecan Spark multimode microplate reader (Tecan Trading AG, Switzerland). This assay was performed four times with four replicates per treatment.

#### Virulence Assay

The effect of naringenin on the virulence of Pb1692 was assessed by measuring the severity of disease symptoms on *Zantedeschia aethiopica* (calla lily) and *Solanum tuberosum* (potato).^32^ Fully extended young leaves of calla lily were surface sterilized in 0.5% sodium hypochlorite for 20 min. The leaves were then washed twice with DDW and dried under a laminar flow hood. Leaf discs, approximately 20 mm in diameter, were excised and placed on Petri dishes containing MS medium. Pb1692 cultures grown in liquid LB 16 h, 28 °C, were diluted to 1 × 10^8^ CFU/mL in minimal media (MM) with or without naringenin (control), and incubated on a shaker (2 h, 28 °C, 150 rpm). The leaf discs were inoculated by pipetting at the center of 10 μL of the suspensions. The infected leaf discs were incubated at 28 °C without shaking. Disease severity was evaluated as the percentage of decayed tissue relative to the total disc area after 15 h. The infection assays were repeated three times with 20 replicates.

For potato, the bacteria were diluted to 5 × 10^6^ CFU/mL in distilled water with or without 0.5–2 mM naringenin and incubated on a shaker (2 h, 28 °C, 150 rpm). After 2 h of incubation, 10 μL of the bacterial suspensions were used to inoculate the sterilized potato tubers by piercing with a pipet. The inoculated potatoes were incubated without shaking at 28 °C for 48 h. Disease severity was assessed by weight as the percentage of rotten tissue after 48 h of exposure. The experiment was repeated 4 times with four replicates for each treatment.

#### Plant Cell Wall Degrading Enzymes

The bacterial production of plant cell wall-degrading enzymes (PCWDEs) was assessed semiquantitatively.^33^ Wells were made using a number 2 cork-borer. Single colony of Pb1692 was grown for 16 h at 28 °C, washed, and resuspended in fresh LB as described above. Bacterial concentrations were adjusted to 5 × 10^6^ CFU/ml and incubated under shaking (8 h, 28 °C, 150 rpm) with or without the addition of naringenin (0.5–2.0 mM). After 8 h, all the treatments were centrifuged (14,000 rpm, 5 min) and the supernatant was transferred to a new Eppendorf tube, of which 20 μL were used to fill the previously prepared wells. Following 16–18 h without shaking, pectate lyase (Pel) and polygalacturonase (Peh) plates were treated with 4 N HCl and left for 10 min, after which clear haloes were visible. The protease enzyme plates (Prt) were left for 48 h of incubation for visible haloes. The activity of the enzymes was determined based on the halo area. The assay was repeated 3 times with 8 replicates per treatment.

#### Motility Assay

Swimming- and swarming-motility assays were done as described.^34^ A single colony of Pb1692 was incubated ON in 4 mL of LB (28 °C, 16 h) with continuous shaking (150 rpm). After 16 h, bacteria were washed and adjusted to 5 × 10^6^ CFU/ml in fresh LB containing naringenin (0.5–2.0 mM). The treatments were incubated for another 2 h at 28 °C with shaking (150 rpm). The swimming-motility plates contained 1% tryptone, 0.5% NaCl, and 0.3% agar, while the swarming-motility plates contained 1% tryptone, 0.5% NaCl, 0.5% dextrose, and 0.5% agar. Both motility plates were stably inoculated with 2 μL in the center of the plate. The plates were incubated (24 h, 28 °C), without shaking. Motility was measured by the bacterial cell migration distance from the point of inoculation. The assay was repeated 3 times with 8 replicates per treatment.

### Gene Expression Analysis

#### RNA Extraction and cDNA Preparation

Pb1692 was grown for 16 h, 28 °C in LB liquid medium under continuous shaking at 28 °C. Two μL were inoculated to fresh 4 mL liquid LB without or with naringenin (0.5- 2.0 mM), and incubated (8 h, 28 °C, 150 rpm), under continuous shaking. After 8 h, 2 mL samples were transferred to an Eppendorf tube and centrifuged (5 min, 14,000 rpm), and the pellet was used for RNA extraction. The GENEzol Reagent (Geneaid, Shijr District, New Taipei City, Taiwan) was used according to the manufacturer’s instructions with slight modifications. The bacterial pellet was lysed with 1 mL of GENEzol, and incubated for 30 min at RT. Then, 100 μL of 2-bromo-3-chloropropanee (Thermo Scientific, Acros) was added, and the mixture was vigorously shaken for 10 min. The samples were centrifuged (15 min, 12,000*g*, 4 °C). The upper phase was transferred into a new 1.5 mL Eppendorf, and an equal volume of 2-propanol was added and mixed gently by inverting 20 times. The samples were kept ON at −20 °C, then centrifuged (10 min, 12,000*g*, 4 °C), the supernatant was discarded carefully, and pellet was resuspended in 4 M lithium chloride and incubated (3 h, −20 °C). After centrifuging (12,000*g* for 15 min, 4 °C) the pellet was washed twice with 75% ethanol and air-dried for 10 min under chemical hood. RNA was resuspended by adding 30–50 μL of DNase/RNase free water. The extracted RNA was used to prepare cDNA using a cDNA synthesis kit (Applied Biosystems, Foster City, CA, USA). The cDNA reverse-transcription reaction was performed using a programmable thermal controller (MJ Research, St. Bruno, PQ, Canada) programmed to one cycle at 42 °C for 30 min, followed by activation at 95 °C for 2 min, after which the cDNA was stored at −20 °C for future use.

#### Quantification of mRNA by qRT-PCR

Real-time PCR was conducted to quantify the transcripts level of specific virulence genes.^35,36^ Briefly, the primers were designed using the National Center for Biotechnology Information (NCBI) primer BLAST software (http://www.ncbi.nlm.nih.gov/tools/primer-blast/), 100–120 bp in size and melting temperature of 60 °C, with a difference of less than 5 °C for each primer pair (SI, Table S2). To exclude nonspecific binding, primer sequences were analyzed by BLAST (using NCBI BLAST software) against the database for the genus *Pectobacterium.* The primer mixture for qRT-PCR contained 5 μL of Fast SYBR Green Master Mix (Applied Biosystems) and 0.8 μL (5 μM) of each forward and reverse primer. A total of 3.4 μL (17 ng) of cDNA was added to each well so that the total reaction mixture would be 10 μL for each well. Reactions were performed using a Step One Plus Real-Time PCR system (Applied Biosystems), with the following cycling parameters: holding stage, 95 °C for 20 s; cycling stage, 40 cycles of 95 °C for 3 s and 60 °C for 30 s,and melting curve stage, 95 °C for 15 s, 60 °C for 1 min, and 95 °C for 15 s. The data were analyzed by the comparative CT (ΔΔCT) method, with expression normalized to the expression of the reference gene *ffh*, as described by.^37^

#### ROS Measurement

Measurement of reactive oxygen species (ROS) was performed.^38,39^ A single colony of Pb1692 was grown ON in a 50 mL falcon tube and 10 mL of LB, at 28 °C under continuous shaking (150 rpm). Then, 1 mL of bacteria was transferred to 1.5 mL of Eppendorf and centrifuged (5 min, 10,000 rpm, RT). Supernatant was discarded, and the pellet was resuspended in fresh LB containing naringenin (0.5–2.0 mM). LB without naringenine and DMSO served as controls. All treatments were grown at 28 °C for 3 h, under dark conditions with continuous shaking, and then centrifuged (10,000 rpm, 5 min), and the supernatant was discarded. The samples were washed twice with phosphate buffer saline (PBS, pH = 7.2), and resuspended in fresh LB containing 20 μM of 2′,7′-dichlorofuorescein diacetate (DCFDA) dye in PBS. Then 200 μL of each sample were transferred to a 96-well plate and incubated (1 h, 28 °C), under dark conditions. After the incubation, the medium consisting of 2′,7′-dichlorodihydrofluorescein diacetate (DCFDA) was decanted, and 200 μL of fresh PBS was added to each well. The fluorescence intensity was measured using a Tecan Spark multimode microplate reader (Tecan Trading AG, Switzerland) at excitation and emission wavelengths of 485 and 528 nm, respectively. The image of the same plate was taken with In Vivo Imaging System (IVIS) Lumina II imaging system software (PerkinElmer, USA), to analyze the florescence intensity of all treatments visually with wide lens E camera type IS1330N6337, Andor, iKon.

#### Polyphenol Oxidase Activity in Response to Naringenin

To further explore the oxidative potential of naringenin on Pb1692, the activity of polyphenole oxidase (PPO) was assayed.^40–42^ A single colony of Pb1692 was grown (ON, 28 °C) in 4 mL of LB in a 15 mL tube, under continuous shaking (150 rpm). Two milliliters of the cultures were transferred to an Eppendorf tube and centrifuged (3 min, 12,000 rpm, RT). Supernatant was discarded, and the pellet was resuspended in fresh LB, and measured at OD 600 nm. Next, sterile conical flasks (200 mL) were used to grow 100 mL of Pb1692 5 × 10^6^ CFU/ml in LB containing 100 μM of copper sulfate (CuSO_4_), with or without naringenin. The flasks were incubated for 48 h at 28 °C, with continuous shaking (150 rpm), transferred to 50 mL tubes, and centrifuged at 10,000 rpm for 10 min at 4 °C. This step was repeated 4 times with PBS for washing, after which the pellet was resuspended in 8 mL of 100 mM potassium phosphate buffer (pH= 6.5). Lysis of bacterial cells was executed by ultrasonication using 6–8 bursts of 5 min in ice-cold water, each at 100% power, with 5 min intervals. The cell debris were removed by centrifugation 12,000*g*, 4 min, at 4 °C, and cell free supernatant collected and concentrated using vivaspin 20 mL (30,000 MWCO) (Vivascience AG, 30625 Hannover, Germany), and centrifuged, 3000*g*, 5 min, at 4 °C. This eluent was used as crude PPO preparation, in a 96-well microtiter plate, with a total reaction mixture of 110 μL, 50 μL aliquots of crude enzyme, 50 μL of 5 mM 2,6-dmethoxyphenol (DMP) in potassium phosphate buffer, pH = 7.0, and 10 μL of 1 mM CuSO_4_. After 10 min of incubation at 30 °C, microplate was recorded at 468 nm wavelength, in a Tecan Spark multimode microplate reader (Tecan Trading AG, Switzerland).

#### Membrane Leakage

Bacterial conductivity assay was conducted according to with some modifications.^43^ A single colony of bacterial cells was first cultured overnight in 10 mL of fresh LB under continuous shaking 150 rpm at 28 °C. Next morning the culture was centrifuged at 4000*g* for 5 min, the supernatant was discarded, and pellets were resuspended with sterile distilled water (DW). The bacterial cells were washed 3 times with distilled water and finally resuspended with 2 mL of sterile distilled water. The OD was measured at 600 nm. Naringenin (0, 0.5 mM, 1 mM and 2 mM) was added to the bacterial suspensions 10^8^ CFU/ml with DMSO and DW served as positive controls, and 2 mM of naringenin in DMSO without bacteria served as a negative control. After 3 h, the conductivity was measured using a conductivity meter. The experiment was repeated twice with 3 replicates each time.

#### Computational Studies

Molecular docking of naringenin and *S*-adenosyl methionine (SAM) were conducted on a recently published homology model of ExpI, derived from the crystal structures of TofI from Pantoea stewartii and EsaI from Burkholderia glumae.^44^ The ExpI model features two binding sites: one for the acyl chain (AC) of the acylated acyl-carrier protein (ACP) and another for the SAM substrate. Docking of SAM and naringenin to the SAM site was carried out by means of the Glide Standard Precision method as implemented in Schrodinger’s software.^45,46^ Two docking experiments were conducted for each ligand, in the absence and presence of AC. When AC was considered, its position within the binding site was determined by aligning its structure to J8-C8, an acyl-HSL synthase inhibitor of the TofI protein (PDB 3P2H) that occupies the AC site. The grid box for SAM and naringenin docking was centered on the SAM binding site. During the docking of SAM, distance constraints were applied between the carboxyl group of AC near the sulfur atom and the positively charged amine group of SAM, in accord with the proposed reaction mechanism for AHL synthase.^47^ Based on the docking results, two distinct binding poses for SAM and two for naringenin in the presence and absence of AC were selected for subsequent Molecular Dynamics (MD) simulations (a total of eight poses).

MD simulations were conducted using the Desmond simulation package by Schrödinger LLC.^48^ Prior to simulations, the proteins were prepared using the Protein Preparation Wizard as implemented in Maestro. Subsequently, the systems were solvated using the TIP3P water model in cubic box and neutralized with Na^+^ ions, and then NaCl was added to achieve a final salt concentration of 0.158 M. The OPLS4 force field parameters were used in all simulations. Long-range electrostatic interactions were computed with the particle mesh Ewald method, using a cutoff radius of 9.0 Å for Coulomb interactions. Nonbonded forces were calculated using an r-RESPA integrator, the short-range forces were updated every step, and the long-range forces were updated every three steps.

The time step employed for all simulations was 2 fs. The systems were first minimized using the steepest descent, followed by the LBFGS method until the maximum force on any atom was below 1.0 kcal/mol/Å. Next, the system was relaxed using a standard relaxation process consisting of five restrained short simulations, each run for 100 ps: (1) an NVT simulation with Brownian dynamics at 10 K, small timesteps, and restraints on solute heavy atoms; (2) an NVT simulation at 10 K using a Langevin thermostat for 12 ps with fast temperature relaxation, velocity resampling every 1 ps, and solute restraints; (3) an NPT simulation at 10 K and 1 atm for 12 ps with a Langevin thermostat and barostat, fast temperature relaxation, slow pressure relaxation, velocity resampling every 1 ps, and solute restraints; (4) an NPT simulation at 300 K and 1 atm for 12 ps under similar conditions; (5) an NPT simulation at 300 K and 1 atm for 24 ps with a normal pressure relaxation constant. The production phase was performed under NPT conditions at 301.15 K and 1 bar. Each simulation was run for 1000 ns. Pressure was controlled using the Martyna–Tuckerman–Klein chain coupling scheme with a coupling constant of 2.0 ps, while the temperature was controlled using the Nosé–Hoover chain coupling scheme. Nonbonded forces were computed using an r-RESPA integrator,^49^ with short-range forces updated at every step and long-range forces updated every three steps. The trajectories were saved at 4.8 ps intervals for analysis.

During the MD simulations, restraints were applied to the two hydrogen bonds between AC and the backbones of Phe102 and Arg101 in ExpI. These restraints were introduced to compensate for the absence of ACP in our system, ensuring that AC remained correctly positioned within the binding site. In the case of SAM, an initial simulation was performed with three restraints: two on the hydrogen bonds between AC and the backbone of Phe102 and Arg101, and one between the carboxyl group of AC (positioned near the sulfur atom) and the positively charged amine group of SAM, following the proposed reaction mechanism for AHL synthase. Subsequently, the binding free energy for these simulations were calculated using the Prime MM-GBSA method. The frame with the lowest binding free energy in each case was then selected as the input for a second MD simulation, this time using only two restraints, those maintaining the hydrogen bonds between AC and the backbones of Phe102 and Arg101.

The Simulation Interaction Diagram tool in the Desmond MD package was used to analyze ligand–protein interactions and behavior in the final set of eight trajectories. Simulation stability was monitored by assessing the root mean square deviation (RMSD), using the initial frame as a reference. The Prime MM-GBSA method was used to estimate ligand binding free energies from the MD trajectories using the thermal_mmgbsa.py script of the Prime/Desmond module of the Schrodinger suite.

#### Docking to RecA

A homology model of RecA for *Pectobacterium brasiliense* was developed based on its sequence as downloaded from the NCBI (National Center for Biotechnology Information) databases and the crystal structure of RecA from *E. coli* RecA (PDB code 3CMT;^50^). The two proteins share a sequence identity of 89.6%. Homology modeling was performed using the Modeler program.^51–53^ The stereochemical quality of the resulting model was evaluated using ProCheck^54^ and Prosa,^55^ yielding satisfactory results: 93.5% and 5.8% of residues were found to be within the allowed and generously allowed regions of the Ramachandran plot, respectively, Prosa’s Z-score was found to be −7.31, and the template-target RMSD was found to be 1.1 Å.

The binding region of RecA is classified into four binding pockets based on their locations and physiological functions:^56,57^ pocket A (ATP binding); Pocket B (unknown); pocket C (dsDNA binding); and pocket D (ssDNA binding). Thus, compounds were docked into all sites with known function (A, C, D) using the Glide-SP method as implemented in Schrodinger’s software. The results are shown in SI, Figure S4.^56^

#### Data Analysis

One-way analysis of variance (ANOVA) was made using JMP Pro Software. If ANOVA indicated a significant difference (*p* < 0.05), a Tukey–Kramer HSD multiple-comparison test was performed. Graphs were constructed using GraphPad Prism Version 8.3.0 (GraphPad, San Diego, CA, USA). Fiji ImageJ (http://fiji.sc/Fiji) was used to measure areas of virulence and enzymatic assays.

## Results

### Effect of Naringenin on Growth of *P. brasiliense* (Pb1692)

The effect of naringenin on the growth of Pb1692 was evaluated by using a minimum inhibitory concentration (MIC) assay. The bacteria were exposed to increasing concentrations (0.5–6.0 mM) of naringenin. Growth curves are presented in Figure 1A. Concentration of naringenin that inhibited bacterial growth by less than 50% were considered as nonlethal concentrations. While none of the concentrations used completely inhibited bacterial growth, concentrations above 2 mM appeared to have an increased inhibitory effect that was limited by poor solubility of the compound. Accordingly, to further explore the effects of naringenin on bacterial virulence we used the concentrations 0.5, 1.0, and 2.0 mM in most of the experiments, cell counts were also performed using dilution plating method (SI, Figure S2B).

**Figure 1:**
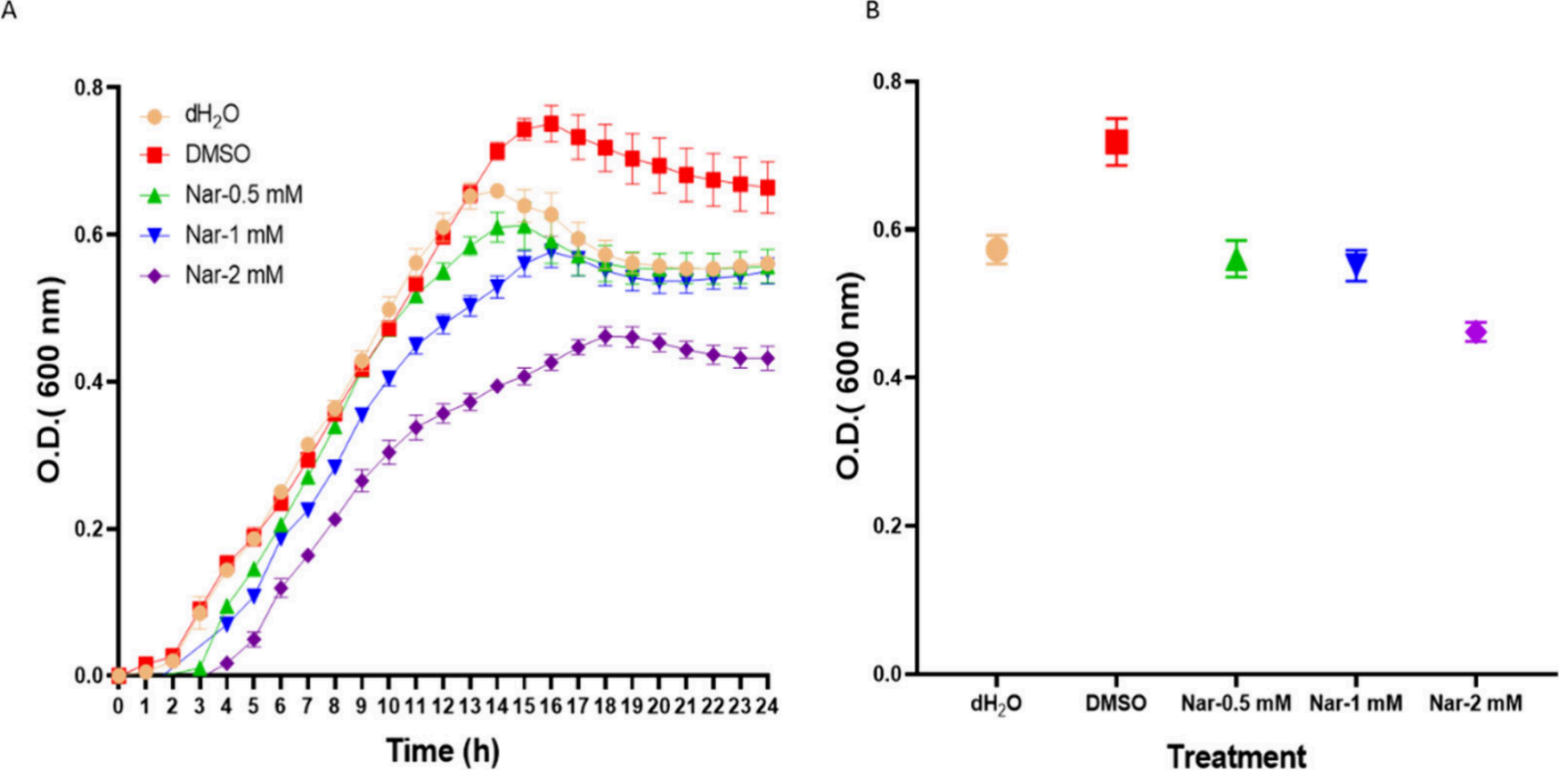
*Pectobacterium brasiliense* Pb1692 growth curves following exposure to naringenin over 24 h. Pb1692 cultures were treated with naringenin (0.5–2.0 mM), water (dH_2_O) or DMSO (0.8%) as controls (A). Bacterial growth occurred at 24 h following exposure to Naringenin. The data points and standard error mean (SEM) represent 4 replicates per treatment from three independent experiments (B).

### Effects of Naringenin on AHL Production

The effect of naringenin on AHL production was studied in Pb1692, using *E. coli* reporter strain pSB401. This strain quantitatively reports the presence of AHL molecules by producing a luminescence signal. The intensity of the signals was proportional to the increasing concentrations of naringenin, as presented in Figure 2. Accordingly, exposure of pSB401 to the supernatant of Pb1692 treated earlier with naringenin concentration of 1 mM or 2 mM, displayed significantly reduced luminescence intensity over 18 h period showing at 18 h 50% and 95% reduction respectively, as compared to the dH_2_O-treated control or 0.8% DMSO, which was used as solvent (Figure 2A,B). Exogenous AHL (eAHL) (200 nM, 20 μL) was used as a positive control and produced RLU values similar to those of the dH_2_O control. To analyze the direct effect of naringenin on AHL production in Pb1692 after 18 h, we have quantified 3-oxo-C6-HSL and 3-oxo-C8-HSL using LC-MS/MS in the supernatant of the bacterium cultures. The results revealed three- and five- fold reduction in 3-oxo-C6-HSL and 3-oxo-C8-HSL respectively, in response to all experimental concentrations of naringenin (0.5–2 mM) with no significance difference between all treatments (Figure 2C–F). Accordingly, to test the direct inhibitory effect on AHL production in the LC-MS/MS analysis, only the lowest naringenin concentration (0.5 mM) was presented in the chromatogram.

**Figure 2:**
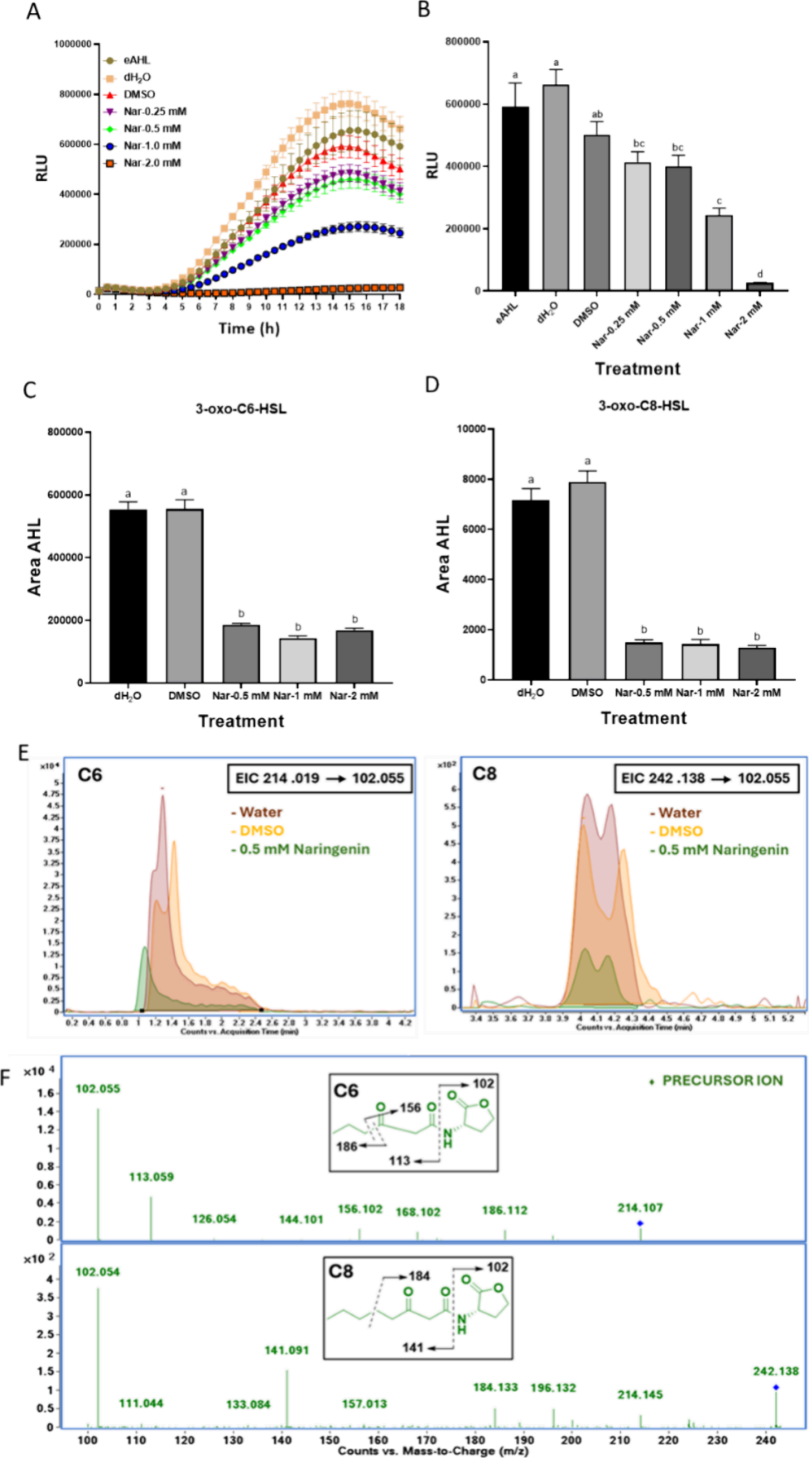
Luminescence intensity (RLU = LU/OD600) of *Escherichia coli* pSB401 induced by eAHL (positive control) and supernatants from *Pectobacterium carotovorum* Pb1692 treated with naringenin (0.5–2 mM), water, or 0.8% DMSO (controls) was measured every 0.5 h over 18 h (A). Bar graph shows RLU at 18 h for increasing naringenin concentrations (B). Quantitative levels of 3-oxo-C6-HSL (C) and 3-oxo-C8-HSL (D) in Pb1692 suspensions with naringenin are shown. Extracted ion chromatograms (EICs) for 3-oxo-C6-HSL and 3-oxo-C8-HSL ([MH^+^]: 214.019 and 242.138 to 102.055 fragment ion) display retention time (*x*-axis) and signal intensity (*y*-axis) (E). High-resolution fragmentation spectra by collision-induced dissociation, with precursor ions marked by blue diamonds, are shown for both AHLs and metabolites (F). Data represent the SEM of 8 replicates per treatment from two independent experiments. Statistical differences were analyzed by one-way ANOVA with Tukey–Kramer HSD. Bars that are labeled with a different letter are considered significantly different (*p* < 0.05).

### Direct ExpI Inhibition by Naringenin Using *Chromobacterium violaceum* as Reporter

The QS-negative *E. coli* strain DH5α was used to explore direct inhibition of ExpI by naringenin. The plasmid pGEM-*expI* was expressed in a QS negative DH5α strain, under the control of the T7 promoter.^44^ The transformed strain DH5α/pGEM *expI* (applied in the middle dashed circle) was able to efficiently produce AHL under control conditions, as observed by strong violacein synthesis by reporter CV026 (Figure 3). In contrast, DH5α/pGEM control (without ExpI) did not produce detectable levels of AHL, while naringenin application to the central paper disc (2 mM, 30 μL) strongly inhibited the synthesis of AHL by ExpI as demonstrated by the minute levels of violacein pigment (Figure 3). Since 2 mM naringenin hardly affected the growth of DH5α+ExpI or CV026, shown by bacterial counts (SI, Figure S2A,C), the inhibitory effect was evidently the result of direct inhibition of ExpI, as no other components of the QS machinery are present in DH5α.

**Figure 3:**
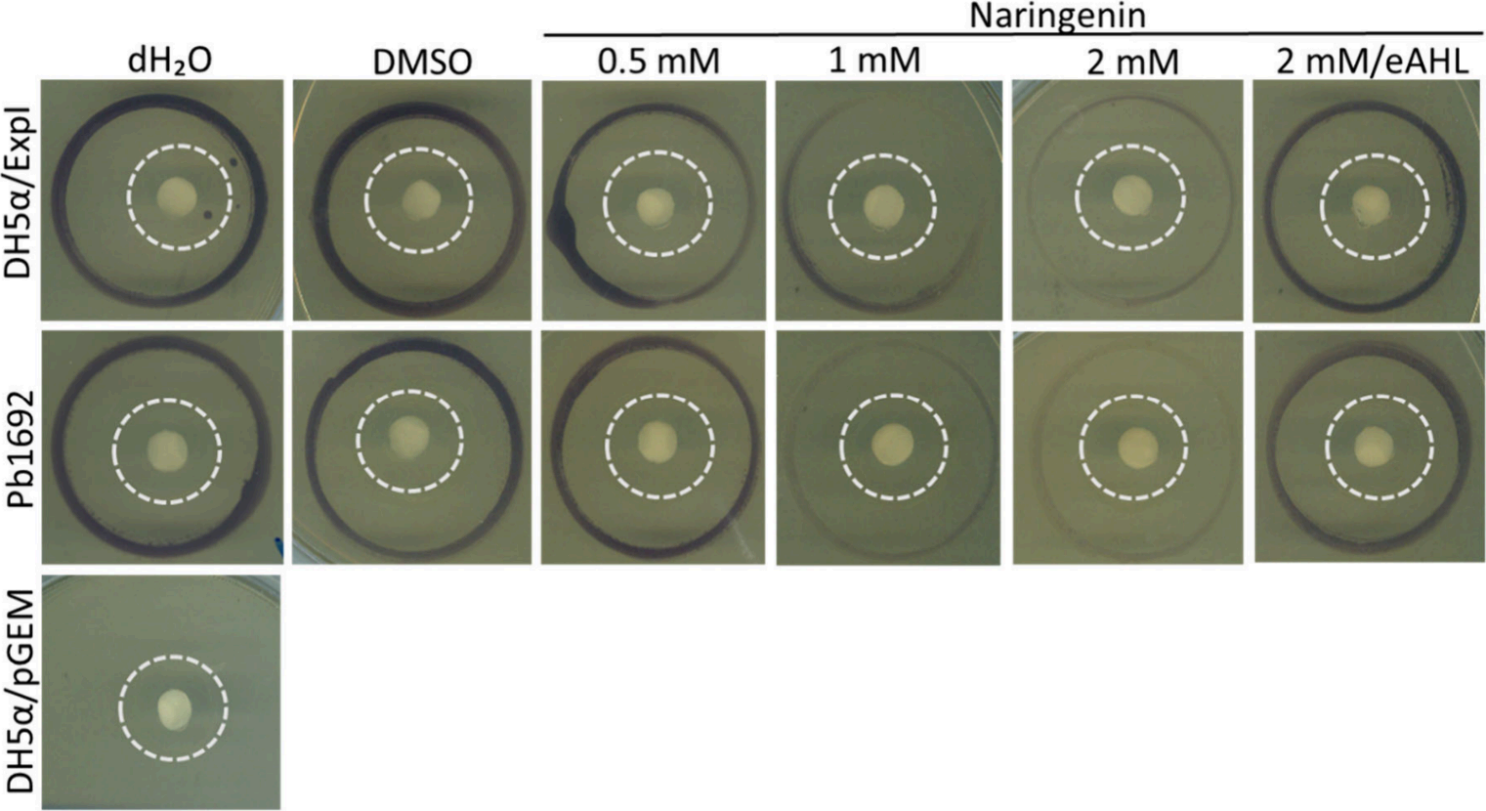
Visualization of violacein (purple pigment) production by CV026 in response to AHLs from DH5α/pGEM *expI* (top panel, inner circle), Pb1692 (WT) (middle panel, inner circle), and DH5α/pGEM (negative control, bottom panel). Central paper discs were treated with 30 μL of dH_2_O, 0.8% DMSO, or naringenin (0.5–2.0 mM). For rescue, 30 μL of 500 nM 3-oxo-C6-HSL (eAHL) was added after 2 mM naringenin. The outer circle (3 cm diameter) contained CV026; the inner circle (1.5 cm diameter) contained test strains.

### Effects of Naringenin on Biofilm Formation and Motility

The ability of plant-pathogenic bacteria to survive and persist in the environment is largely dependent on their ability to colonize their hosts and form biofilms. The effect of naringenin on the inhibition of biofilm formation was tested using naringenin (0.5–2 mM). Pb1692 was grown in yeast extract containing tryptone for 72 h after which biofilm formation was significantly reduced at concentrations higher than 1 mM and by up to 33% reduction at 2 mM (Figure 4A).

**Figure 4:**
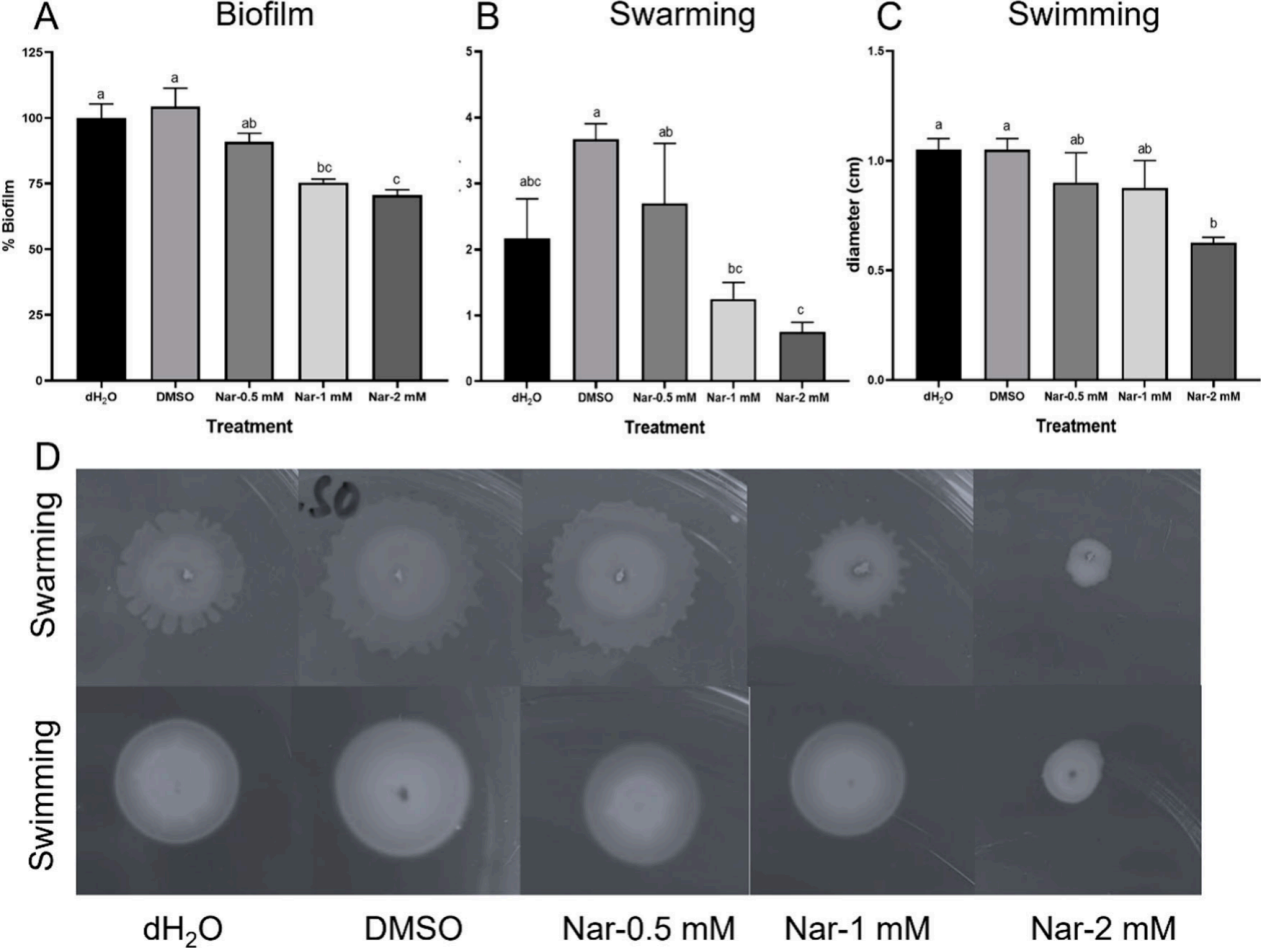
Swarming motility (A) and swimming motility (B) of Pb1692 after treatment with naringenin (0.5, 1, and 2 mM), dH_2_O, or 0.8% DMSO (controls). Representative images of swarming and swimming plates after 24 h at 28 °C (C). Biofilm formation in liquid yeast extract medium after 72 h at 28 °C, quantified by absorbance of crystal violet at 550 nm (D). Each bar represents mean ± SEM; motility assays: 3 independent experiments, 8 replicates per treatment (*n* = 24); biofilm assays: 2 independent experiments, 8 replicates each (*n* = 16). Statistical differences were analyzed by one-way ANOVA with Tukey–Kramer HSD; bars with different letters indicating significant differences (*p* < 0.05).

Another important determinant of virulence in colonization of plant surfaces by plant-pathogenic bacteria including *Pectobacterium* is motility.^58^ Swimming and swarming motility of Pb1692 were assessed to determine the effect of naringenin on motility. The results indicate that exposure of Pb1692 to naringenin affected both modes of motility in a different manner, while motility was generally reduced upon exposure to 2 mM naringenin, the effect on swarming was more complicated displaying a slight enhancement at lower naringenin concentration and upon exposure to DMSO (Figure 4B,C).

### Effect of Naringenin on the Activity and Secretion of PCWDEs

Plant cell wall-degrading enzymes such as pectate lyase (Pel), protease (Prt), and polygalacturonase (Peh) are crucial components of *Pectobacterium* virulence. These enzymes play a key role in initiating soft-rot symptoms. A semiquantitative enzymatic activity assay,^33^ is presented in (Figure 5A–C). Bacterial cultures that were exposed to increasing concentrations of naringenin (0.5–2 mM), displayed reduced PCWDEs activities relative to dH_2_O or DMSO controls.

**Figure 5:**
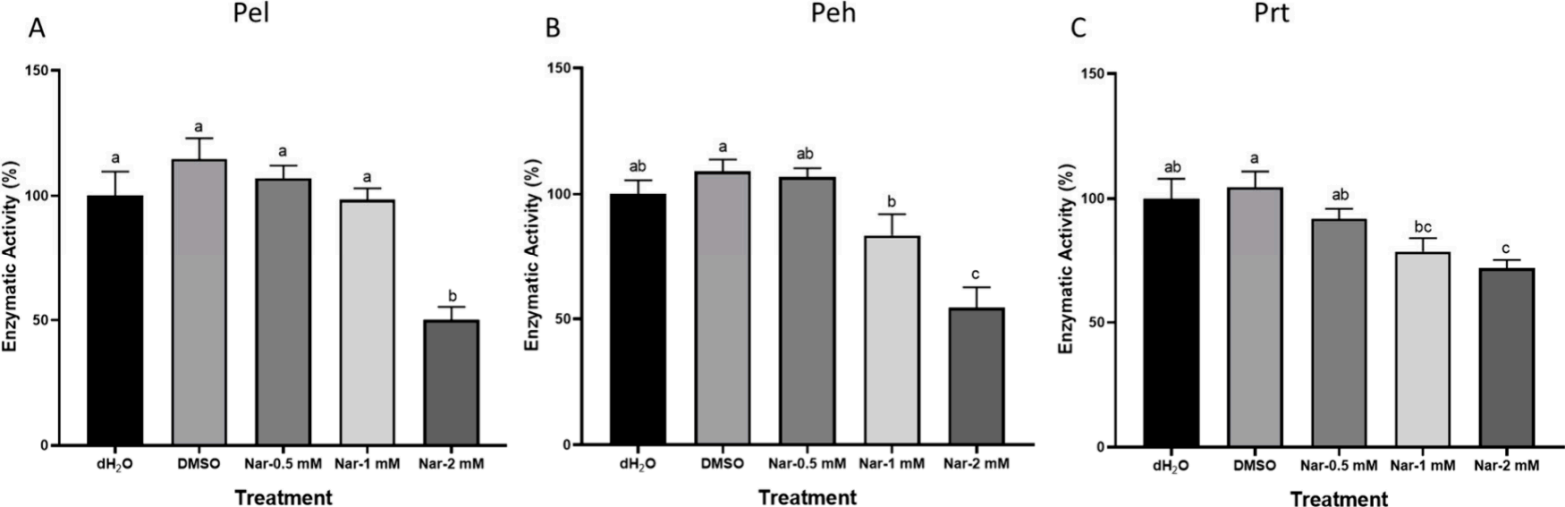
Effect of naringenin concentrations (0.5–2 mM) on pectate lyase (Pel) (A), polygalacturonase (Peh) (B), and protease (Prt) (C) activities in Pb1692. Bars show mean ± SEM (% of dH_2_O control, *n* = 16). One-way ANOVA and Tukey–Kramer HSD were used to analyze differences. Different letters indicate significant differences (*p* < 0.05).

### Effect of Naringenin on Expression of Selected Genes

The effect of naringenin on the relative expression of genes associated with the QS system, motility, and PCWDE-related virulence in Pb1692 was determined using qPCR. The expression levels were evaluated following exposure of bacterial cultures to 0.5–2 mM of naringenin for 8 h. The genes are roughly categorized as QS-system genes (*expI*, *expR*), QS-regulated genes, mainly PCWDE (*pel*, *peh*, and *prt*), and motility related genes linked to bacterial attachment and colonization of the host (*motA*, *fliA, and flhC*). At 1 mM concentration, naringenin significantly suppressed the expression of QS-related genes with *expI* (AHL synthase) suppressed by 4-fold whereas *expR* (response regulator) suppressed by 5-fold when compared with the dH_2_O and 0.8% DMSO control treatments (Figure 6). Relative expression of PCWDEs was also significantly reduced at 1 mM naringenin but not at 2 mM (*pel,* and *prt*) while *peh* expression was dose dependent. A 4- to 5-fold reduction was observed for *motA* and *fliA* when compared to dH_2_O or DMSO, while the expression of the master regulator of flagellar synthesis and function, *flhC*, showed an approximately 10-fold reduction at 1 mM.

**Figure 6:**
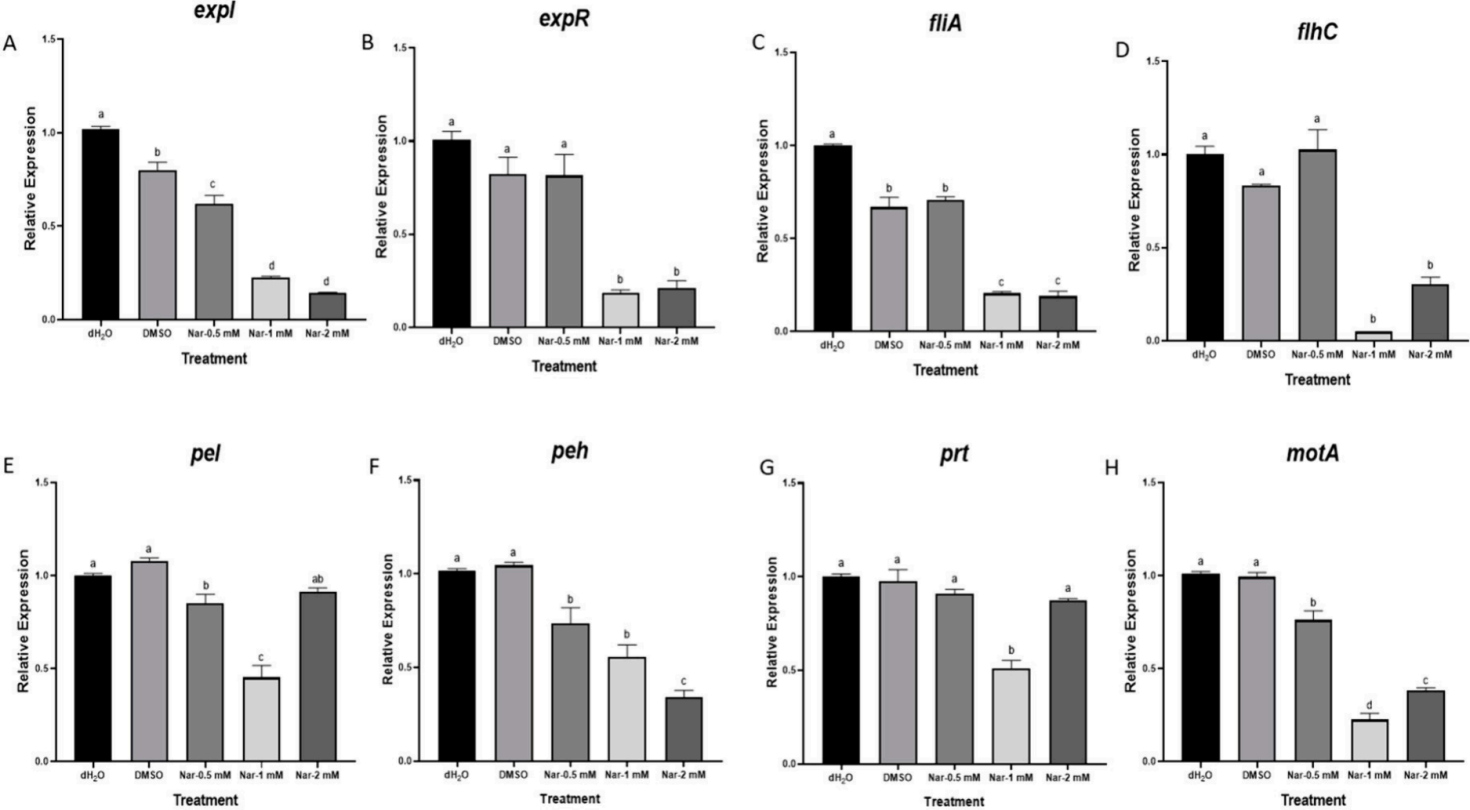
Effect of naringenin (0.5, 1, and 2 mM) on expression of quorum sensing (*expI*, and *expR*), PCWDE (*Pel*, *Peh*, and *Prt*) and motility (*motA*, *flhC*, and *fliA*) genes in *Pectobacterium brasiliense* Pb1692 measured by qPCR after 8 h growth in LB at 28 °C. dH_2_O or 0.8% DMSO were used as controls. Bars show means + SE of 6 replicates per treatment with 4 biological repeats. Different letters indicate significant differences (*P* < 0.05; *n* = 24).

### Effect of Naringenin on Pb1692 Infection in Calla Lily and Potato

Infection assay results reflect the interplay between pathogen virulence and host defense mechanisms. Effective disruption of virulence regulation typically results in reduced disease symptoms. Disease severity was recorded in two common hosts of *Pectobacterium*, *Z. aethiopica* (calla lily), and potato tubers,^32^ upon exposure to naringenin (0.5–2 mM). Tissue decay of potatoes following infection was almost blocked even at 0.5 mM naringenin (Figure 7A). A dose-dependent reduction in disease symptoms severity was also observed in maceration of calla lily leaf discs, with negative correlation between naringenin concentrations and macerated leaf area. Exposure of Pb1692 to 2 mM naringenin reduced symptoms by about 90% in comparison to the controls (M9 and DMSO) (Figure 7B).

**Figure 7:**
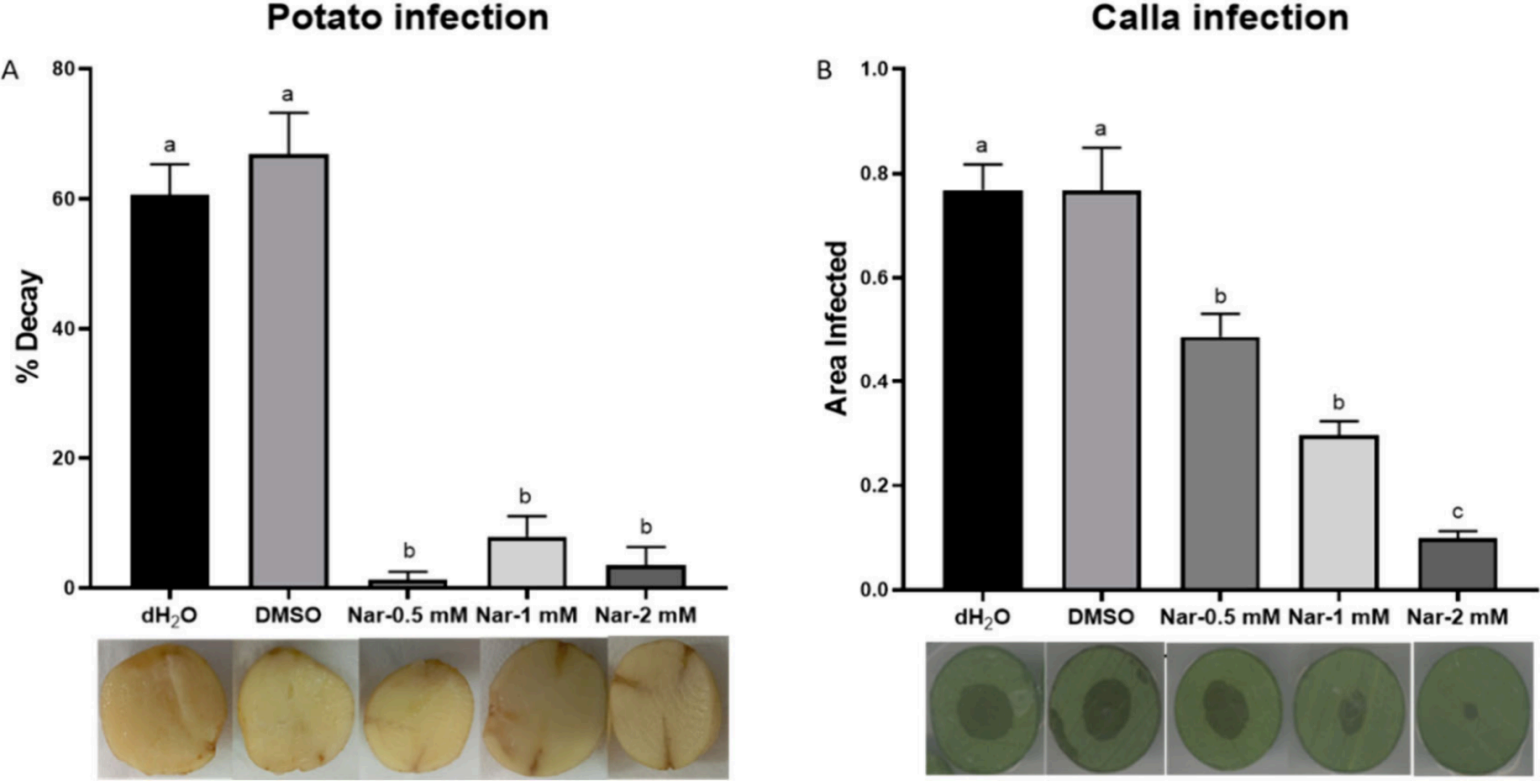
Effect of naringenin (0.5–2 mM) on decay of potato tubers (A) and calla lily leaf discs (B) infected by Pb1692. dH_2_O in minimal media (M9) and 0.8% DMSO served as the controls. Bars show mean ± SEM of percent tissue decay or infected area from two independent experiments (8 replicates/treatment). One-way ANOVA with Tukey–Kramer HSD; different letters indicate significant differences (*p* < 0.05).

### Activation of Reactive Oxygen Species and Stress Response in Pb1692

Naringenin has been reported in the cellular generation of ROS under increasing concentrations of naringenin (Han and Lee, 2022). The gene *recA* responsible for DNA damage repair and SOS response, displayed significantly reduced expression upon treatment with 2 mM naringenin (Figure 8A). This reduction was not observed at lower concentrations of naringenin. A DCFDA dependent ROS measurement assay kit was used, as well as IVIS imaging Lumina II imaging system software (PerkinElmer, USA), to analyze the florescence intensity of all treatments visually with wide lens E camera type IS1330N6337, Andor, iKon. DCFDA was added to Pb1692 following naringenin treatment, showing higher fluorescence at 2 mM naringenin (Figure 8B). Addition of naringenin to the media did not show spontaneous ROS (SI, Figure S8). In contrast to the reduction in *recA* expression polyphenol oxidase activity following exposure to 2 mM naringenin was increased by more than 2-fold relative to control (up to 0.8% DMSO), indicating enhanced PPO activity upon exposure (Figure 8D).

**Figure 8:**
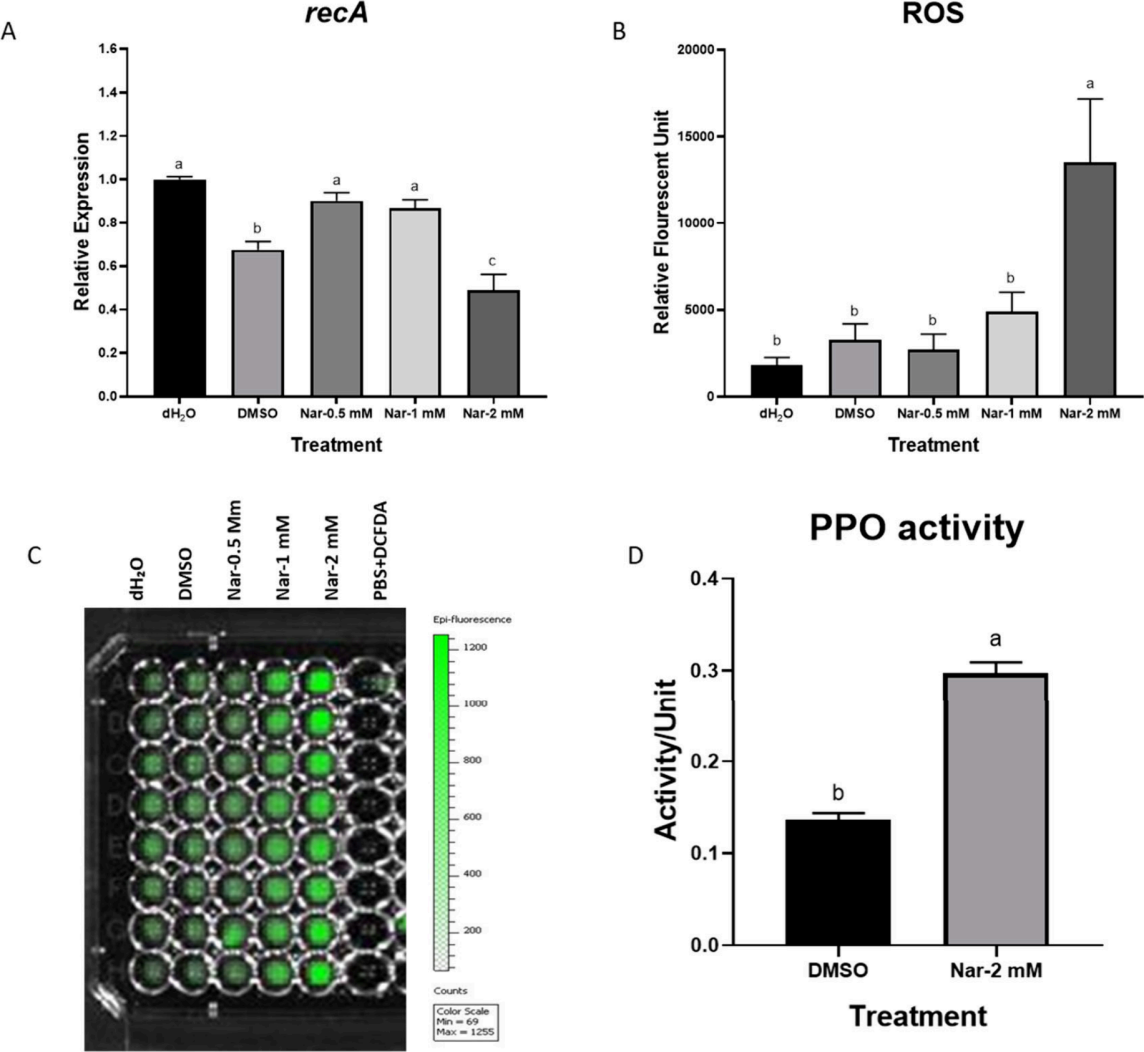
Expression level of RecA (A) ROS formation (B), and PPO activity (D) in *Pectobacterium brasiliense* Pb1692 after exposure to naringenin (0.5–2 mM). dH_2_O or 0.8% DMSO served as controls. Bars show mean + SE from three independent experiments (8 replicates/treatment). IVIS fluorescence image of ROS in 96-well plates (C). One-way ANOVA with Tukey–Kramer HSD was used for (A,B). Student’s *t* test was used for (D). Different letters indicate significant differences (*p* < 0.05).

### Polyphenol Oxidase Activity Following Exposure of Pb1692 to Naringenin

The PPO activity of Pb1692 was tested here as a means of bacterial defense mechanism against oxidative stress. The results indicated enhanced PPO activity relative to control (0.8% DMSO) or water upon exposure to 2 mM of naringenin (Figure 8D).

### Membrane Damage

Membrane leakage was studied by measuring the electrical conductivity of the suspension medium.^43^ The results showed a significant increase in membrane damage upon exposure to naringenin, suggesting that it interferes with Pb1692 membrane integrity (SI, Figure S3).

### Computational Studies

In order to obtain atomic-scale insights into the binding mode of SAM and naringenin in the SAM binding site of ExpI and to evaluate the relative binding free energies of these ligands, we employed molecular docking simulations to identify reliable poses followed by microsecond-long MD simulations to refine them and MM-GBSA calculations to score them. To ensure that the computational studies reflect, as much as possible, the physiological state of this protein, docking and MD simulations were performed both in the presence and in the absence of AC, which interacts with SAM (but not with naringenin) to produce the signal molecule AHL.

Multiple, repeated MD simulations of SAM in the absence of AC were initiated from two different poses obtained from molecular docking, and in all, the ligand detached from its binding site. Thus, for these cases, the trajectories could not be analyzed, and binding free energies could not be evaluated. However, the presence of AC stabilized SAM in its site throughout the entire simulation initiated from one binding mode (pose 1) and throughout the first 310 ns when starting from the second binding mode (pose 2), allowing for the calculation of binding free energies through the MM-GBSA procedure. In the case of naringenin, the ligand remained within its binding site throughout all four simulations. These observations are well supported by the RMSD plots presented in Figure S5 of the Supporting Information.

Figure 10 presents the simulation interaction maps for SAM and naringenin, respectively, whereas Table 1 provides the calculated binding free energies of the two ligands as predicted by MM-GBSA.

**Table 1:**
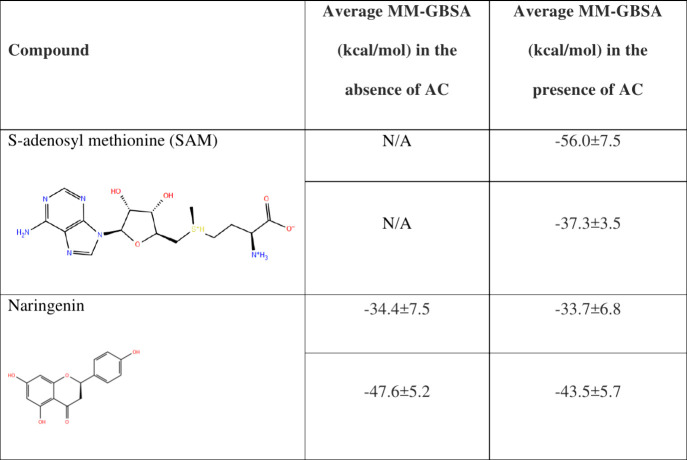
Average Binding Free Energies from the MM-GBSA Procedure, Derived from Desmond MD Trajectories, for SAM and Naringenin in the Absence and Presence of AC

Based on the results of the MD simulations and the data presented in Table 1, SAM is unable to maintain a stable binding mode within the ExpI binding site in the absence of AC, yet the presence of AC stabilizes the ligand within the binding site. In contrast, naringenin is able to stably bind ExpI either in the absence or the presence of AC. Furthermore, the presence of AC does not seem to greatly affect the binding free energy of this ligand (Figures/Videos S6A,B, S7A,B).

Analyzing the simulation interaction diagram between SAM and ExpI in the presence of AC (Figure 9), reveals that the majority of interactions are formed with Trp34 (π–cation interactions), Arg101 (π–cation and hydrogen bond), and Ile142 (hydrogen bond) with additional interactions formed with other, primarily polar residues, some through water molecules. These residues are known to play a crucial role in the binding site.^59^

**Figure 9:**
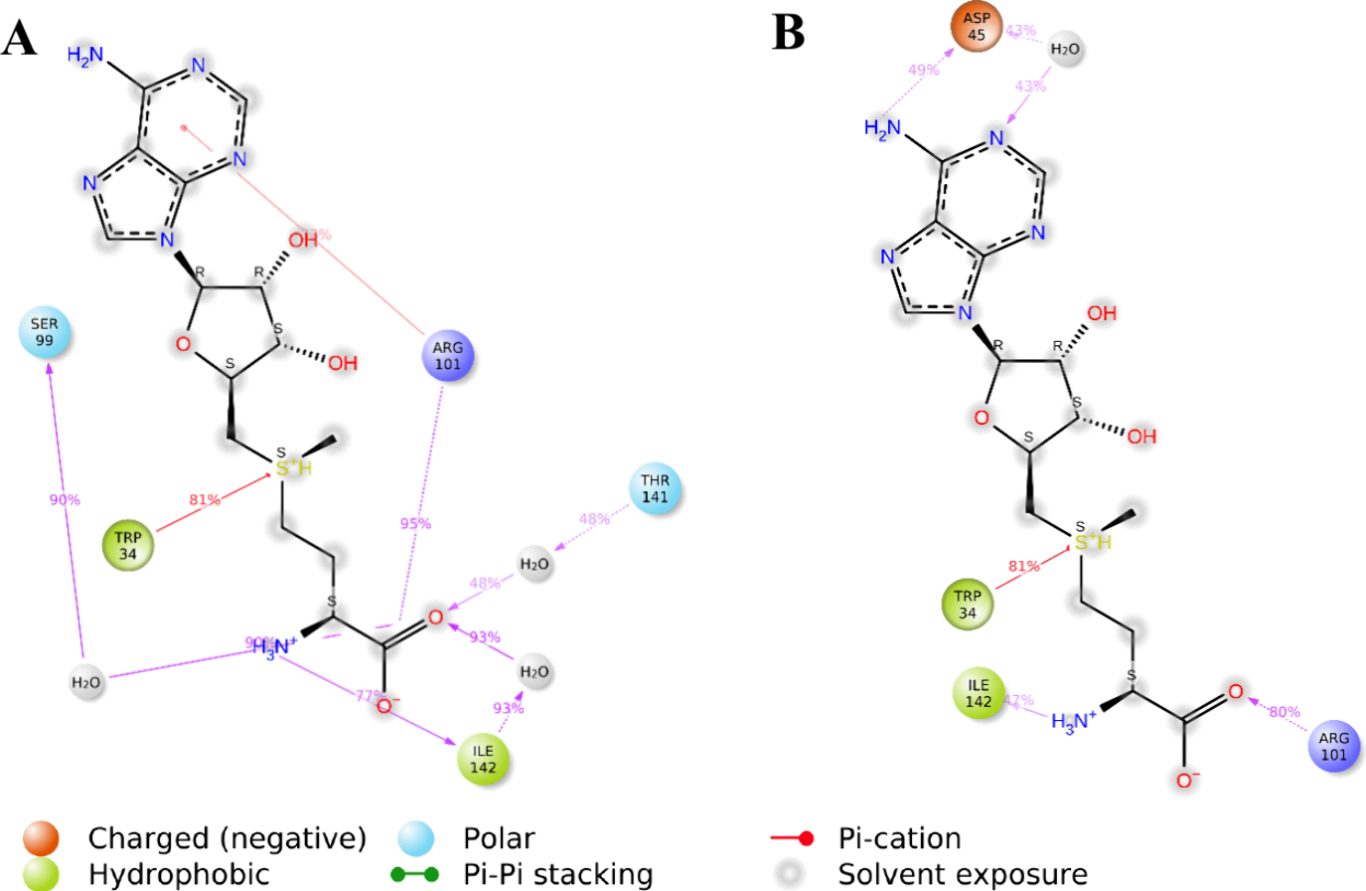
Interactions between SAM and ExpI during the two MD simulations, initiated from two distinct initial poses in the presence of the acyl chain (AC). During the simulations the position of the AC was restrained via two hydrogen bonds to the backbone of Phe102 and Arg101. (A) Interaction map for the trajectory initiated from Pose 1. (B) Interaction map for the trajectory initiated form Pose 2. Note: it is possible to have interactions with >100% as some residues may have multiple interactions of a single type with the same ligand atom. For example, the Arg side chain has four H-bond donors that can all hydrogen-bond to a single H-bond acceptor. For a hydrogen bond to be considered valid, the distance between the hydrogen atom and the acceptor atom (H···A) must be less than 2.8 Å, the angle between the donor atom, the hydrogen atom, and the acceptor (D–H···A) must exceed 120.0°, and the angle formed by H···A–B must be greater than 90.0°. In face-to-face pi–pi interactions, the maximum distance between the centroids of the rings is 4.4 Å with a maximum angle of 30° between the ring planes. For edge-to-face pi–pi interactions, the maximum distance between the centroids of the ring is 5.5 Å, again, with a maximum angle of 30° between the ring planes. In pi–cation interactions, the maximum distance between the center of the cation and the centroid of the ring is 6.6 Å, and the maximum angle between the normal to the plane of the ring and the line between the cation center and the ring center is 30°.

A similar analysis for naringenin (Figure 10) reveals distinct interaction patterns between the ligand and the protein under different conditions. When naringenin was simulated alone, its primary interactions occurred with Trp34, regardless of the initial pose, with interaction frequencies of 28% and 23%. Additionally, strong hydrogen bonding was observed with Glu43 (58%) and Val104 (49% and 22%), while Phe82 and Ile150 also contributed to the binding. These interactions led to a stable binding mode, where a combination of hydrophobic and polar interactions helped anchor the ligand within the binding site.

**Figure 10:**
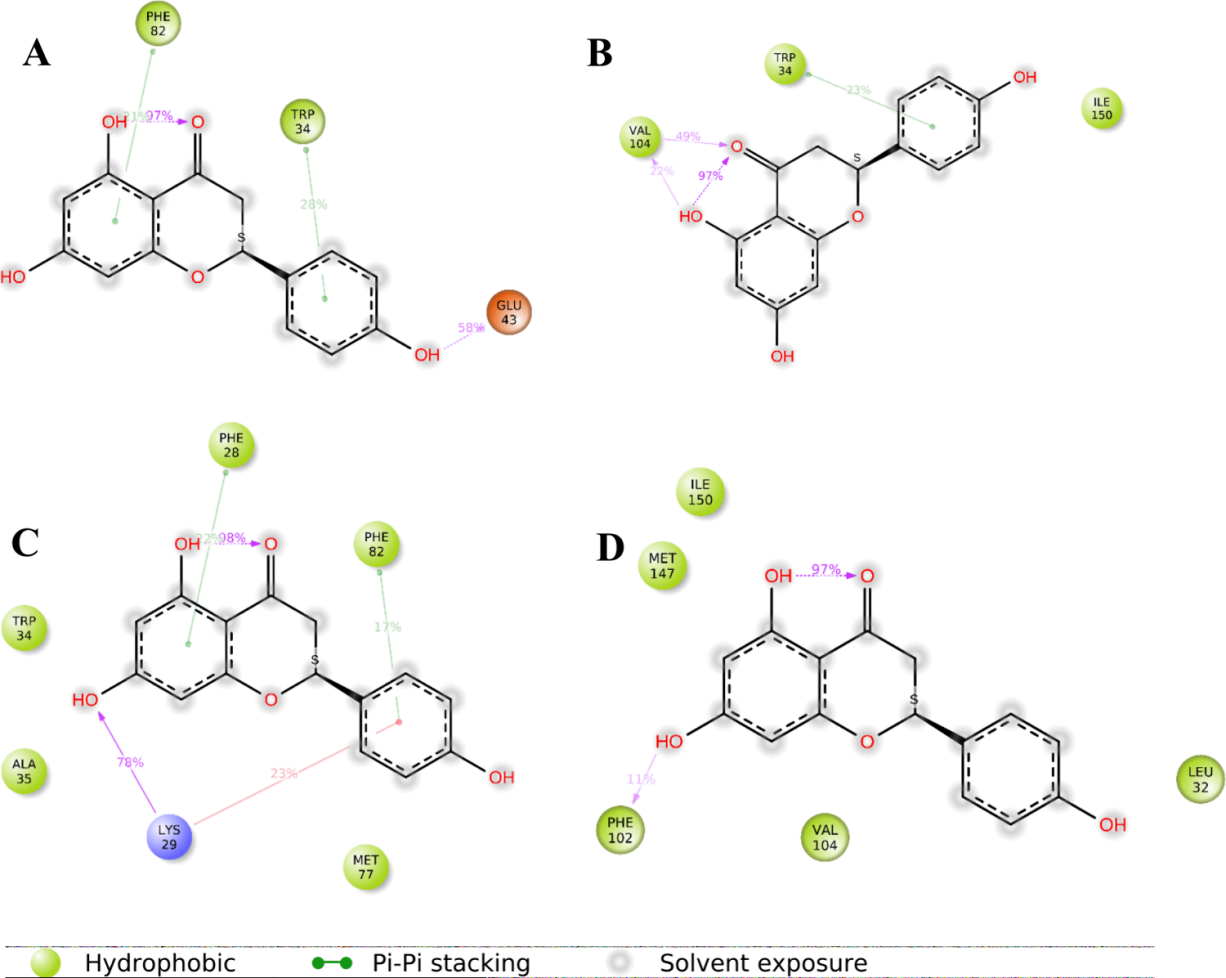
Interactions between naringenin and ExpI during the two MD simulations, initiated from two distinct initial poses in the absence (A,B) and presence (C,D) of an acyl chain (AC). During the simulations the position of the AC was restrained via two hydrogen bonds to the backbone of Phe102 and Arg101. (A) Note: it is possible to have interactions with >100% as some residues may have multiple interactions of a single type with the same ligand atom. For example, the Arg side chain has four H-bond donors that can all hydrogen-bond to a single H-bond acceptor.

In contrast, when naringenin was simulated in the presence of AC, a shift in its interaction profile was observed. The ligand formed a strong hydrogen bond with Lys29 (78%), suggesting a possible rearrangement within the binding pocket. Furthermore, new interactions with Phe28, Phe102, and Phe82 emerged, indicating an alternative binding mode that could influence the ligand stability and affinity. This shift in interactions highlights the dynamic nature of naringenin’s binding behavior and suggests its potential adaptability within the active site. However, despite these variations, Trp34 remained a consistently interacting residue across all simulations, emphasizing its critical role in naringenin binding.

## Discussion

The quorum sensing (QS) machinery has emerged as a promising target for virulence modulation in Gram-negative plant pathogens over the past decades.^60,61^ QS inhibitors can modulate bacterial virulence through multiple regulatory pathways, including signal molecule biosynthesis and detection, gene expression regulation, and QS-dependent phenotypic traits.^8^ However, naturally occurring QS inhibitors frequently exhibit complex, multifaceted mechanisms of action. Our investigation of the citrus flavanone naringenin as a potential QS inhibitor in *P. brasiliense* 1692 revealed intriguing mechanistic insights. While this compound has been identified as a substrate of the AcrAB-TolC efflux system, suggesting that subinhibitory concentrations of this compound may compromise cellular homeostasis leading to its active exclusion from the cell, its cellular mode of action has not been fully considered.^62^ Several studies have reported naringenin as an antimicrobial agent against *E. amylovora,* a devastating pathogen responsible for fire blight disease on pear and apple trees.^18^

Here, the effect of naringenin on the virulence of Pb1692 was investigated. At first, in line with previous results, a concentration that had a less than 50% effect on bacterial cell growth was calibrated. This level of growth inhibition did not affect virulence related phenotypes in Pb1692, as previously confirmed by the simultaneous use of ciprofloxacin, an antibiotic that inhibits growth, but not virulence.^28,63^ The QS machinery and its major protein, AHL synthase (ExpI), is one of the major regulators of virulence in Pectobacteria. The signaling molecules 3-oxo-C6-HSL and 3-oxo-C8-HSL, are essential for host infection, PCWDEs production, flagellar regulation and swimming motility.^6^

Hence, naringenin’s impact on bacterial signaling and AHL production was evaluated using complementary approaches: two biosensor systems and a direct LCMS/MS quantification of 3-oxo-C6-HSL and 3-oxo-C8-HSL. The luminescence-based biosensor strain pSB401, which responds to nanomolar concentrations of 3-oxo-C6-HSL,^24^ demonstrated significant downregulation of AHL production by Pb1692 at naringenin concentrations of 1 mM and higher. The reporter strain CV026 produces violacein in an AHL-dependent manner. Here it was used to detect AHL production by wild-type Pb1692 and *E. coli* DH5α containing the ExpI of Pb1692 (pGEM *expI*). Naringenin (1–2 mM, 30 μL) inhibited violacein production in Pb1692 as expected, whereas in DH5α, a strain that harbors only pGEM *expI* and lacks other QS components, the inhibition strongly supported the direct interference of naringenin with ExpI activity. This direct effect was further validated by LCMS/MS analysis, which confirmed significantly lower levels of both 3-oxo-C6-HSL and 3-oxo-C8-HSL in Pb1692 culture supernatants following naringenin treatment, even at 0.5 mM a concentration that has no effect on cell growth. Collectively, the findings indicate that naringenin functions as a QS inhibitor by directly targeting ExpI, rather than through interference with other components of the QS regulatory network. Similar to our results, naringenin has been associated with reduced production of QS signaling, including the specific inhibition of AHLs 3-oxo-C12-HSL and C4-HSL molecules in *P. aeruginosa.* ^64^

Biofilm formation and motility are two interconnected virulence traits that are largely regulated by quorum sensing (QS) machinery. Biofilm is responsible for bacterial attachment to biotic or abiotic solid surfaces.^65^ It also protects bacteria against harsh environmental conditions and antimicrobial compounds.^65,66^ Here, 0.5 mM of naringenin were sufficient to significantly reduce biofilm formation relative to control, a response that has been reported in both Gram-negative and Gram-positive bacteria.^67,68^ Similar examples were provided for *Streptococcus mutans* and *Vibrio harveyi*, Gram-positive and Gram-negative bacteria, respectively.^67,69^ In *S. mutans*, exposure to 100 and 200 μg/mL naringenin (0.364 mM and 0.730 mM) concentrations that are in the same range as in our experiments (0.5 mM), increased surface hydrophobicity, thereby decreased bacterial adhesion and biofilm maturation.^67^ In *V. harveyi*, 100 μg/mL of naringenin significantly reduced biofilm formation and suppressed the expression of genes related to the type three secretion system (TTSS).^68^ Biofilm was also reduced by 60–70% in *Vibrio cholerae*, at 50 μg/mL (0.18 mM), and swimming motility by 3-to-6- fold in *V. cholerae* strains VC87 and N16961 respectively.^70^ In *Salmonella typhimurium* biofilm formation was not inhibited upon naringenin treatment.^69^

Motility is an important factor employed by plant pathogenic bacteria during host colonization. Swarming motility is a multicellular surface movement on solid media, that requires hyperflagellation, while swimming is characterized as individual movement in liquid media, driven by a rotating flagellum.^71^ Exposure of Pb1692 to naringenin significantly reduced both motility types at 2 mM with a stronger effect on swarming. In cyanobacteria,^72^ or *Ralstonia solanacearum,* ^73^ naringenin had no effect on motility, but in *Salmonella typhimurium* naringenin differentially regulated flagellar operons and inhibited motility.^69^ Naringenin at 4 mM reduced swarming and twitching motility in *P. aeruginosa* PAO1, as well as microcolony confluence. This effect was attributed to the failure to establish compact cell-to-cell attachment, which aligns with our findings.^74^

PCWDEs secretion is regulated by QS and is the most recognized virulence trait associated with SRP.^75^ Exposure of Pb1692 to naringenin decreased PCWDEs Pel, Peh, and Prt secretion in a dose dependent manner up to 3 mM, the limit of the compound’s solubility in our experiments. These findings were reflected as reduced disease symptoms in potato tubers and calla lily leaf discs. Similar decrease in disease severity was reported upon exposure of *Pectobacterium* to other flavonoids such as coumaric acid, salicylic acid, vanillin and catechol suggesting that this arsenal of plant derived compounds is an evolutionary mechanism by which different plant species may interfere with bacterial regulation systems to lessen virulence.^35,36^

The expression patterns of virulence related genes may shed light on the involvement of specific genes in the response to naringenin. To study the expression patterns of the motility genes, *fliA, flhC*, and *motA*, qPCR was employed showing downregulation at 1 or 2 mM naringenin, similar to the responses in *Herbaspirillum seropedicae*, a Gram-negative rhizosphere bacterium,^76^ and *S. typhimurium* LT2, where transcriptome profile revealed that 17 genes involved in flagellar and motility were repressed in the presence of naringenin.^69^

The response of QS genes to naringenin largely varied between the species. While *Vibrio cholera,* ^70^ *Sinorhizobium meliloti,* ^77^ and *Azorhizobium caulinodans,* ^78^ displayed upregulation of QS-related genes, *P. aeruginosa* PAO1 displayed significantly reduced expression of QS genes (i.e., *lasI*, *lasR*, *rhlI*, *rhlR*, *lasA*, *lasB*, *phzA1*, and *rhlA*), and virulence.^64^ Naringenin also intensely reduced the production of the AHLs *N*-(3-oxododecanoyl)-l-homoserine lactone (3-oxo-C12-HSL) and *N*-butanoyl-l-homoserine lactone (C4-HSL), which is driven by the *lasI* and *rhlI* gene products, respectively.^64^ Our results showed downregulation of the key QS genes *expI* and *expR*, which well correlated with the reduced levels of AHL upon treatment with 0.5, 1, or 2 mM naringenin. One mM naringenin also downregulated the expression of the QS dependent genes *pel*, *peh*, and *prt*. Surprisingly, at 2 mM, the trend was reversed and the expression was upregulated to the level of the control treatments. We ruled out the hypothesis that this pattern was a result of poor solubility, and based on previous studies we postulated that a cellular stress response, or oxidative stress, may have caused the upregulation of *pel.* ^79–81^ The discrepancy between increased *pel* gene expression and reduced Pel enzymatic activity (at 2 mM naringenin) likely reflects post-transcriptional regulation. It may be due to the well-characterized RsmA-*rsmB* system in *Pectobacterium*. RsmA represses translation of PCWDE mRNAs, while *rsmB* antagonizes this effect. Quorum sensing (QS) signals regulate the balance between these factors. As naringenin inhibits QS, it may increase RsmA activity and reduce *rsmB*, leading to translational repression and mRNA degradation despite elevated transcription.^82^ In *Erwinia chrysanthemi*, environmental stress upregulated the expression of *pel* and *prt*.^83–86^ In line with that, the effect of 2 mM naringenin on reactive oxygen species (ROS) formation was evaluated, as well as *recA* expression. RecA mediates cellular responses to DNA damage by activating SOS repair genes, and its function becomes particularly important under oxidative stress that results in DNA damage.^87^ It facilitates LexA autocleavage to activate SOS response genes.^88,89^ Exposure of Pb1692 to naringenin triggered a strong ROS response at 2 mM, a response that was also observed in *P. syringae* and *E. coli* upon treatment with naringenin.^90,91^ Despite the observed increase in ROS production and potential DNA damage, our results revealed an unexpected downregulation of *recA* expression, suggesting an impairment of the DNA repair mechanism. This paradoxical response has been previously documented in treatments with other compounds: both *p*-coumaric acid and 1,4-naphthoquinone induced similar downregulation patterns.^91,92^ Various phenolic compounds, including *p*-coumaric acid,^92^ and curcumin,^56,93,94^ have demonstrated dual effects on RecA, modulating both its transcriptional expression and protein activity. The compounds gallic acid, *p*-coumaric acid, and curcumin reduced *recA* expression in *Staphylococcus aureus*,^95^ *Listeria monocytogenes*,^92^ and in *E. coli*,^93^ respectively. Moreover, these compounds exhibited an additional mode of action by directly inhibiting the RecA protein activity. To check for potential interactions between naringenin and RecA, we docked this compound as well as curcumin and ciprofloxacin, two known RecA inhibitors, into three binding sites of the Pb1692 RecA homology model (ATP site, dsDNA binding site, and ssDNA binding site).^56^ The results are shown in Figure S4 and suggest that naringenin may favorably bind the ATP binding site and to a lesser extent the ssDNA binding site,^56^ and thus function as a competitive inhibitor of ATP in its binding pocket. Furthermore, naringenin’s docking scores are similar to the docking scores of curcumin and ciprofloxacin. This hypothesis, however, needs further verification.

Bacterial cell membranes are another factor that may be damaged by exposure to elevated levels of naringenin.^73,96^ Our results suggest that membrane integrity is an additional mechanism by which naringenin interferes with the Pb1692 competence. This membrane disruption may be at least in part involved in the oxidative stress encountered by Pb1692. The induction of polyphenol oxidases (PPOs) as a line of defense against ROS, is part of this stress response. PPOs also known as laccases, belong to a group of copper enzymes that oxidize phenolic compounds using oxygen as an electron acceptor.^97,98^ Bacterial PPOs generally protect cells against oxidative stress as well as phenolic compounds, a common mechanism employed by plants against pathogenic bacteria.^98^ These enzymes are involved in resistance to copper and UV as well as in the tolerance to toxic compounds such as ROS and phenols.^97^ Upon exposure to naringenin, specific PPO activity was upregulated, encoded by a PPO gene similar in sequence (87% identity, Figure S1) to the gene reported in *P. atrosepticum*.^97^ The presence of PPO gene in Pb1692 and its sequence were analyzed and confirmed by PCR (Figure S9). Antioxidant enzymes present in bacteria such as superoxide dismutase (SOD) and catalase are controlled by transcriptional regulators such as oxyR or RpoS (general stress response regulators) that respond to oxidative stress.^99–101^ QS inhibition and ROS accumulation in Gram-negative bacteria stem from the interconnected roles of QS in regulating oxidative stress defenses and virulence during the infection of host plants. Curcumin is recognized as strong inhibitor of QS and virulence in *P. aeruginosa*.^102^ Curcumin application increased the level of intracellular ROS, and downregulated the antioxidant enzymes catalase by 4.3-fold, peroxidase by 5.4-fold, and superoxide dismutase (SOD) by 3.7-fold .^100^ Thus, curcumin, an inhibitor of QS also strongly disrupts ROS detoxification, leading to intracellular ROS accumulation. The findings in *P. aeruginosa* indicate a link between QS inhibition and ROS accumulation.

Finally, to gain better insight into the interactions of naringenin with the active site of ExpI, MD simulations were conducted for the first time in the presence or absence of the acyl carrier chain (AHL precursor) in its distinctive binding site on the same enzyme. The analysis was executed in comparison to SAM, the natural ligand of AHL. The results of the MD simulation clearly showed that naringenin is a better binder of ExpI in the absence of AC, while in its presence, the binding values of the two compounds are similar (those of SAM are more dependent on the initial pose of the simulation). Taken together, these findings suggest that naringenin may effectively compete with SAM for the ExpI’s binding site by this inhibiting AHL synthesis. This interference with AHL production downregulates the transcription of *Pectobacterium* virulence genes, which are controlled by the QS system.

Yet despite naringenin’s favorable antibacterial effects, its practical usage in agricultural applications is challenging because of its limited aqueous solubility. To address this shortcoming, several practical strategies could be used. For example, nanoformulations such as polymeric nanoparticles (e.g., chitosan, dextran sulfate) or lipid-based carriers (e.g., liposomes, solid lipid nanoparticles) effectively encapsulate naringenin, improving its solubility and stability. In addition, surfactant-based approaches, including nonionic surfactants (e.g., Tween 80) or micellar solutions, offer additional alternatives to solubilize naringenin at higher concentrations without inducing toxicity. These methods are supported by recent advances in nanodelivery systems, as detailed by Bhia, M. et al., 2021 review.^103^

Alternatively, the structure of naringenin could be modified to develop new analogs with improved aqueous solubility. This approach, however, requires careful structure activity relationship (SAR) analysis to ensure that such modifications do not compromise naringenin‘s ExpI binding. In order to provide preliminary SAR information, we examined the lowest Glide score pose of naringenin in the ExpI site and found it to engage in interactions with binding site residues through the phenyl and carbonyl moieties of its chromanone system while the two hydroxyl moieties of this system as well as the hydroxyl group on the 4-hydroxyphenyl moiety do not participate in specific interactions with binding site residues (Figure S10). Thus, we have computationally designed six naringenin analogs where we “mutated” in turn the carbonyl moiety on the chromanone system as well as the three phenolic groups to hydrogen atoms and dearomatized the two aromatic rings by mutating them to cyclohexane. These six analogs were then docked into the ExpI binding sites and their Glide score examined (Figure S10). We found that Naringenin analogs lacking “interacting moieties” indeed presented poorer docking scores in comparison with the parent compound, whereas the Glide scores of analogs lacking noninteracting moieties were much less affected. This approach could be used to suggest strategic modifications to the structure of Naringenin in order to improve its solubility while maintaining its binding affinity to the ExpI site.

In conclusion, plant-derived phenolic compounds demonstrate multiple antimicrobial mechanisms, simultaneously targeting bacterial virulence and fitness. Flavonoids such as naringenin are well-documented for their broad-spectrum antimicrobial properties. While previous studies have largely focused on their direct bactericidal effects, our work advances the field by demonstrating naringenin’s multimodal action: it simultaneously inhibits quorum sensing (ExpI), disrupts membrane integrity, increases reactive oxygen species (ROS) production, and appears to modulate RecA-dependent DNA repair processes. Collectively, these mechanisms attenuate *Pectobacterium* virulence without imposing strong selection pressure for resistance, aligning with emerging antivirulence strategies that are favored over traditional bactericidal approaches. Additionally, by addressing the solubility challenges of naringenin, our study highlights the critical need to bridge the gap between laboratory efficacy and practical agricultural application-a key step for translating flavonoid-based antimicrobials into real-world use. These findings emphasize the potential of natural flavonoids as antimicrobial agents and underscore the importance of structure–activity relationships in developing effective pathogen control strategies.

## Supplementary Material











## Notes

Bacterial strains used in the study; list of primers used in the study; multiple sequence alignment of polyphemol oxidase (PPO) gene from NCBI *Pectobacterium brasiliense* 1692 (Pb) with PPO from *P. atrosepticum* (Pa) and the PPO sequence from the PCR amplified product of strain Pb1692 (Sq); effect of of naringenin on growth of *Chromobacterium violaceum* (CV026) and *Pectobacterium brasiliense* (Pb1692); effects of naringenin on membrane leakage of *Pectobacterium brasiliense* Pb1692; the RecA homology model structure; root mean square deviation (RMSD) of protein (blue line) and ligand (red line) derived from the MD trajectories; the 1000 ns MD simulation trajectory of naringenin in the ExpI binding site shows that naringenin remains within the binding site throughout the entire simulation; the 1000 ns MD simulation trajectory of naringenin in the ExpI binding site in the presence of AC; the 1000 ns MD simulation trajectory of SAM in the ExpI binding site indicates that SAM detached from the binding site during the simulation; the 1000 ns MD simulation trajectory of SAM in the ExpI binding site in the presence of AC; photo of fluorescence detected by IVIS without bacterial cells; analysis of PCR-amplified product by agarose gel electrophoresis of polyphenol oxidase gene from Pb1692; docking analysis of naringenin analogs in the ExpI binding site; (A) 2D interaction diagram of naringenin with ExpI binding site residues (DOCX)
Video S6A (MPG)
Video S6B (MPG)
Video S7A (MPG)
Video S7B (MPG)
