# Supplementary material for: Multimodal Inhibition of Pectobacterium brasiliense Virulence by the Citrus Flavanone Naringenin
Source: J Agric Food Chem. 2025 Aug 6;73(33):20792–809. doi: 10.1021/acs.jafc.5c04312 (PMC12371876; doi:10.1021/acs.jafc.5c04312)
Supplement: Supplementary file 1 [file jf5c04312_si_001.docx]

**Supporting Information for Publication**

**Multi-modal inhibition of *Pectobacterium brasiliense* virulence by the citrus flavanone naringenin**

**Manoj Pun¹^,^², Ortal Galsurker¹, Netaly Khazanov³, Amy Charkowski^4^, Shani Yelin¹^,^², Zohar Kerem², Michal Weitman^3^, Hanoch Senderowitz*****³, Iris Yedidia*¹**

¹The Institute of Plant Sciences, Volcani Center, Agricultural Research Organization (ARO), Rishon Lezion, Israel

^2^The Robert H. Smith Faculty of Agriculture, Food and Environment, The Hebrew University of Jerusalem, Rehovot, Israel

³Department of Chemistry, Bar-Ilan University, Ramat Gan, Israel

^4^Department of Agricultural Biology, Colorado State university, Fort Collins, CO, United States

*Corresponding authors

Email: [irisy@volcani.agri.gov.il](mailto:irisy@volcani.agri.gov.il)**;** [hsenderowitz@gmail.com](mailto:hsenderowitz@gmail.com)

**Supplementary Figures and tables:**

**Table S1. Bacterial strains used in the study**

| Strain | Genotype^a^ | Reference |
| --- | --- | --- |
| *Escherichia coli* |  |  |
| JM109 (pSB401) | AHL bioluminescent biosensor (pSB401 [Tet^r^, luxR, luxCDABE]) | ^24^ |
| DH5α | *endAI, hsdR17, supE44, thi-1, recA1, gyrA96, ΔlacU169, relA1* | Invitrogen |
| DH5α+*expI* | Amp^R^, pGEMT derivative containing *expI* gene in DH5α | ^44^ |
| *Pectobacterium brasiliense* Pb1692 | Dicot strain isolated from *Solanum tuberosum*, NCBI Accession No. PRJNA31121 | ^102^ |
| CV026 | Mini-Tn5 mutant derived from *C. violaceum* ATCC 31532 HgR, *cvil::*Tn5 *xylE*, Km^R^, plus spontaneous Str^R^, AHL (C4–C8) biosensor. | ^27^ |

^a^ Tet^R^= Tetracycline resistance, Amp^R^= Ampicillin resistance, Km^R^= Kanamycin resistance, Str^R^= Streptomycin resistance

**Table S2.** List of primers used in the study.

| Primer Name | | Sequences | | Use | | Origin |
| --- | --- | --- | --- | --- | --- | --- |
| *expI_F* | TAC AAT AGC GGC AGG CAC TC | | QS signal generator; acyl-homoserine lactone synthase | | Joshi et al. (2016a) | |
| *expI_R* | TGA GAA TCA GGA AGC ATT GGC | |  | | Joshi et al. (2016a) | |
| *expR_F* | TGA GGT CAT GAG ATG TCG CC | | QS transcriptional regulator | | Joshi et al. (2016a) | |
| *expR_R* | TTA TGC CGT CGT AGC GAT CC | |  | | Joshi et al. (2016a) | |
| *pel_F* | CTT CTT CAT GGC CGA TCC CA | | Secretion of pectate lyase | | Joshi et al. (2016a) | |
| *pel_R* | CAA CGG ACT GTG GCT GAT TG | |  | | Joshi et al. (2016a) | |
| *peh_F* | TAC CGC TAC GAG TAC GAC GA | | Secretion of polygalacturonase | | Joshi et al. (2016a) | |
| *peh_R* | GAT CCC ACC AGC TCA CCT TT | |  | | Joshi et al. (2016a) | |
| *ffh _F* | TGG CAA GCC AAT TAA ATT CC | | Signal recognition particle subunit (housekeeping gene) | | Moleleki et al. (2017) | |
| *ffh _R* | TCC AGG AAG TCG GTC AAA TC | |  | | Moleleki et al. (2017) | |
| *pro_F* | GCC GCA CCA AGT CAT TCT AT | | Putative protease | | Moleleki et al. (2017) | |
| *pro_R* | CTT CCA GCG TTT CCA GTA GC | |  | | Moleleki et al. (2017) | |
| *fliA _F* | TAT CAA CAG CGC GAA AGA TG | | Flagellin protein | | Moleleki et al. (2017) | |
| *fliA _R* | ATG CGC TGA GTC ATT TCC TT | |  | | Moleleki et al. (2017) | |
| *fim_F* | GCT TTC ACA CTC GCC ATC TT | | Putative fimbrial protein | | Moleleki et al. (2017) | |
| *fim_R* | GCC TTG CTC TCC GTA CTC AC | |  | | Moleleki et al. (2017) | |
| *motA_F* | TTG CCT ACG GTT TTG TCT CC | | Flagellar motor; chemotaxis protein MotA | | NCBI | |
| *motA_R* | ACA GCG TTT TAC GAC CGA AT | |  | | NCBI | |
| *flhC_F* | ATT GCT GCA AAG GGA TGT TC | | Flagellar transcriptional activator FlhC | | NCBI | |
| *flhC_R* | CCT GTT CAT CCA GCA GTT GA | |  | | NCBI | |
| *recA_F* | CGG CAG CAC TGG GTC AAA TCG A | | DNA repair and maintenance | | NCBI | |
| *recA_R* | GCA CAC GTT TTG CCT TCG CGT T | |  |  | NCBI | |
| *ppo_F* | ATG CTG ATA TAC CCC GAC TGG C | | Polyphenol oxidase  Polyphenol oxidase | | Gorshkov et al. (2017) | |
| *ppo_R* | TAT CAG CCA GAT TAA ACT  TGC CAT AC | |  |  | Gorshkov et al. (2017) | |
|  |  | |  |  |  |  |

**Supplementary Figure 1**

A. CLUSTAL O(1.2.4) multiple sequence alignment at nucleotide level

```
PPO_Pa ATGCTGATATACCCCGACTGGCCTTTGCCAGAAAGTGTGAAGTCTTGCAGTACGACGCGC 60
PPO_Pb ATGCTGATATACCCCGACTGGCCTTTGCCAGAAAGTGTGAAATCTTGTAGTACGACGCGT 60
PPO_Sq --------------CGACTGGCCTTTGCCAGAAAGTGTGAAATCTTGTAGTACGACGCGT 46
                     *************************** ***** ***********

PPO_Pa ATTGGCGGGCATAGTACGTCACCTTATGATTCGCTGAATTTGGGAAATCATGTGGGTGAC 120
PPO_Pb GTTGGCGGACGTAGTGGCGCACCTTATGATTCGCTGAATTTAGGGTGTCATGTGGGCGAT 120
PPO_Sq GTTGGCGGACGTAGTGGCGCACCTTATGATTCGCTGAATTTAGGGTGTCATGTGGGCGAT 106
        ******* * ****    ********************** **   ********* **

PPO_Pa GAGTCCGAACTAGTCAGCGCAAACAGGCAAACGCTGGTGGCGATGGCCGATCTTCCTGCT 180
PPO_Pb GAGCCCGCGCACGTTACCGCAAATCGGCAAGCGCTGGTGGAGATGGCTGGCCTTCCCACT 180
PPO_Sq GAGCCCGCGCACGTTACCGCAAATCGGCAAGCGCTGGTGGAGATGGCTGGCCTTCCCACT 166
       *** ***  *  ** * ******  ***** ********* ****** *  *****  **

PPO_Pa ATGCCGCACTGGTTGGAACAGGTTCATGGTACCGATGTTATTCGCATTGGCGGAGTACTT 240
PPO_Pb ATGCCGCATTGGTTGGAACAGGTGCATGGCACCGATGTGATTCGTCTTGGCGAGACGTCC 240
PPO_Sq ATGCCGCATTGGTTGGAACAGGTGCATGGCACCGATGTGATTCGTCTTGGCGAGACGTCC 226
       ******** ************** ***** ******** *****  ******

PPO_Pa CCATCGTCCGTTTGCGGTGATGCCGCTTATACAGATAAGAAAGGCCAGGTCTGTGCGGTG 300
PPO_Pb CCAACATCCGTTTGTGGTGATGCTGCTTATACGAATAAGAAAGGAAAAGTCTGTGCGGTG 300
PPO_Sq CCAACATCCGTTTGTGGTGATGCTGCTTATACGAATAAGAAAGGAAAAGTCTGTGCGGTG 286
       *** * ******** ******** ********  **********  * ************

PPO_Pa ATGACGGCTGACTGTTTGCCTGTGCTTTTTTGTGCAACCAACGGCGATGAAGTGGCTGCT 360
PPO_Pb ATGACGGCTGACTGCTTGCCCGTGCTTCTTTGTGCAATCAACGGCGATGAAGTCGCTGCC 360
PPO_Sq ATGACGGCTGACTGCTTGCCCGTGCTTCTTTGTGCAATCAACGGCGATGAAGTCGCTGCC 346
       ************** ***** ****** ********* *************** *****

PPO_Pa GCACATGCTGGCTGGCGGGGATTGCACTCCGGGGTGTTGGAAGAAACGTTAGCCTGTTTC 420
PPO_Pb GCTCATGCTGGCTGGCGGGGATTGCACGCGGGGGTATTGGAAGAAACGTTATCCTGTTTC 420
PPO_Sq GCTCATGCTGGCTGGCGGGGATTGCACGCGGGGGTATTGGAAGAAACGTTATCCTGTTTC 406
       ** ************************ * ***** *************** ********

PPO_Pa CGAGCGAAACCTACGCAAATTATGGCATGGCTAGGACCCGCTATCGGGCCTGATGCTTTT 480
PPO_Pb CGTGCACAACCCGAACAAATTATGGCGTGGCTGGGGCCTGCTATTGGGCCTGATGCCTTT 480
PPO_Sq CGTGCACAACCCGAACAAATTATGGCGTGGCTGGGGCCTGCTATTGGGCCTGATGCCTTT 466
       ** **  ****    *********** ***** ** ** ***** *********** ***

PPO_Pa GAAGTCGGCCCAGAAGTTAGGGACGCGTTTATTCAGCATAATGCGTCGGCGGCCTCTGCA 540
PPO_Pb GAGGTCGGTCCTGAAGTCAGGGACGCATTTATTCAGCATGATGCGGCTGCGGCCTCAGCA 540
PPO_Sq GAGGTCGGTCCTGAAGTCAGGGACGCATTTATTCAGCATGATGCGGCTGCGGCCTCAGCA 526
       ** ***** ** ***** ******** ************ ***** * ******** ***

PPO_Pa TTTAGGCCTGAAGGAAACAAATTTTTTGCCGATATCTACCAACTGGCAATTTTACGTTTA 600
PPO_Pb TTTCGGCCTGTAGGAAATAAATTTTTTGCCGATATTTACCAACTGGCAAGTCTGCGTTTA 600
PPO_Sq TTTCGGCCTGTAGGAAATAAATTTTTTGCCGATATTTACCAACTGGCAAGTCTGCGTTTA 586
       *** ****** ****** ***************** ************* * * ******

PPO_Pa CGCGCCGCAGGGATAACGCAAATTTTTGGTGGAAATTCCTGTACTGTGAGCGAGCCTCAC 660
PPO_Pb CGTGCTGCAGGGGTAACGCAAATTTTTGGTGGAAATGCCTGTACTGTGAGCGAGCCTCAC 660
PPO_Sq CGTGCTGCAGGGGTAACGCAAATTTTTGGTGGAAATGCCTGTACTGTGAGCGAGCCTCAC 646
       ** ** ****** *********************** ***********************

PPO_Pa AAATTTTTCTCTTATCGGCGTGATGGCGTGACTGGCCGTATGGCAAGTTTAATCTGGCTG 720
PPO_Pb AAATTTTTCTCTTATCGGCGTGATGGCGTGACTGGCCGTATGGCAAGTTTAATCTGGCTG 720
PPO_Sq AAATTTTTCTCTTATCGGCGTGATGGCGTGACTGGCCGTATGGCAAGTTTAATCTGGGCT 706
       *********************************************************

PPO_Pa ATA-	723
PPO_Pb ATA-	723
PPO_Sq GATA	710
```

**Figure S1:** Multiple sequence alignment of polyphemol oxidase (PPO) gene from NCBI *Pectobacterium brasiliense* 1692 (Pb) with PPO from *P. atrosepticum* (Pa) and the PPO sequence from the PCR amplified product of strain Pb1692 (Sq). The alignment was performed using CLUSTAL O (1.2.4).

**Supplementary Figure 2**

**Figure S2:** Effect of of naringenin on growth of *Chromobacterium violaceum* (CV026) and *Pectobacterium brasiliense* (Pb1692). (**A**) Bacterial cell counts (Log 10 CFU mlˉ¹) of CV026 cells grown in the presence of increasing concentrations of naringenin. Each bar represents mean ± standard error (SE) of 16 replicates per treatment. (**B**) Bacterial cell counts (Log 10 CFU mlˉ¹) of Pb1692 cells grown in the presence of increasing concentrations of naringenin. Each bar represents mean ± standard error (SE) of 12 replicates per treatment. (**C**) Bacterial cell counts (Log 10 CFU mlˉ¹) of DH5α + pGEM ExpI grown in the presence of increasing concentrations of naringenin. Each bar represents mean ± standard error (SE) of 12 replicates per treatment. Bars that are not labelled with the same letter are signiﬁcantly different from each other (*P* < 0.05). One-way ANOVA with post hoc Tukey-Kramer HSD tests was used to analyze differences. Analysis was made by GraphPad Prism 8.0.

**Supplementary Figure 3**

**Figure S3:** Effects of naringenin on membrane leakage of *Pectobacterium brasiliense* Pb1692. Overnight-grown bacteria were washed 3 times in sterile distilled water (DW) and suspended in DW alone (control) or DW containing DMSO or DMSO with naringenin at increasing concentrations. The conductivity of the bacterial suspensions was measured after 3 h.

**Supplementary Figure 4**

**A)**

**C)**

**B)**

**D)**

| Gscore, SP, (kcal/mol) | ADP binding site | dsDNA binding site | ssDNA binding site |
| --- | --- | --- | --- |
| naringenin | -5.36 | -4.13 | -5.09 |
| curcumin_enol_form_stable | -5.06 | -3.29 | -3.19 |
| curcumin_keto_form | -4.71 | -3.32 | -4.909 |
| curcumin_quinone_methide | -3.16 | -3.23 | -4.67 |
| ciprofloxacin | -4.70 | -4.75 | -5.10 |

**Figure S4:** The RecA homology model structure includes three binding sites, namely, the ATP binding site (**A**), the dsDNA binding site (**B**), and the ssDNA binding site (**C**). The ATP binding site (**A**), highlighted in green, is defined by residues Glu69, Lys73, Thr74, Glu97, Tyr104, Gln195, and Asp145, which were used to set the docking grid box. The dsDNA binding site (**B**), shown in purple, consists of residues Arg244, Lys246, Ser173, Arg177, Gln195, and Arg197, used for grid box definition. The ssDNA binding site (**C**), marked in baby blue, includes residues Met198, Lys199, Ile200, Gly201, Thr211, Gly212, Gly213, and Asn214, which were used to establish the docking grid box. (**D**) The Table provides Glide SP docking scores for naringenin, curcumin, and ciprofloxacin across the three binding sites.

**Supplementary Figure 5**

**A**

**B**

**C**

**D**

**E**

**F**

**G**

**H**

**Figure S5**: Root mean square deviation (RMSD) of protein (blue line) and ligand (red line) derived from the MD trajectories. Graphs (A to H) represent systems involving SAM, Naringenin, and AC within the ExpI bunding site, both individually and in combinations.

(**A**) SAM alone, initiated from pose 1. (**B**) SAM alone initiated from pose 2. (**C**) SAM in the presence of AC, iniriated from pose 1. (**D**) SAM in the presence of AC initiated from pose 2. (**E**) Naringenin alone, initiated from pose 1. (**F**) Naringenin alone initiated from pose 2. (**G**) Naringenin in the presence of AC, initiated from pose 1. (**H**) Naringenin in the presence of AC initiated from pose 2.

**Supplementary Figure 6**

**Figure S6A**: The 1000 ns MD simulation trajectory of naringenin in the ExpI binding site shows that naringenin remains within the binding site throughout the entire simulation.

**Figure S6B**: The 1000 ns MD simulation trajectory of naringenin in the ExpI binding site in the presence of AC was conducted while maintaining two restraints to preserve the hydrogen bonds between AC and the backbone of Phe102 and Arg101. Naringenin remained in the binding site throughout the entire simulation. The carbon atoms in Naringenin are highlighted in orange, while those in AC are shown in yellow.

**Supplementary Figure 7**

**Figure S7A**: The 1000 ns MD simulation trajectory of SAM in the ExpI binding site indicates that SAM detached from the binding site during the simulation.

**Figure S7B:** The 1000 ns MD simulation trajectory of SAM in the ExpI binding site in the presence of AC was conducted while maintaining two restraints to preserve the hydrogen bonds between AC and the backbone of Phe102 and Arg101. The presence of AC stabilized SAM in its binding site throughout the entire simulation. The carbon atoms in SAM are highlighted in purple, while those in AC are shown in yellow.

**Supplementary Figure 8**

**Figure S8:** Photo of fluorescence detected by IVIS without bacterial cells. A 96 wells plate of spontaneous ROS generated by naringenin (0.5-2 mM) and controls (LB and DMSO). No spontaneous ROS production was observed.

**Supplementary Figure 9**

**Figure S9:** Analysis of PCR-amplified product by agarose gel electrophoresis of polyphenol oxidase gene from Pb1692. Each PCR mixture was run on a 1.2% agarose gel for 35 min at 100 V. The gel was stained with ethidium bromide and photographed under UV light. Lane 1, 100 bp DNA ladder (100-1500 bp marker); Lane 2, PPO gene amplified from Pb1692; and Lane 3, control reaction mixture without DNA. The expected product size is 723 bp.

**Figure S10:** Docking analysis of naringenin analogs in the ExpI binding site. (A) 2D interaction diagram of naringenin with ExpI binding site residues. (B) Naringenin analogs designed in this work. In analogs 1, 2, and 3 the hydroxyl group was replaced by a hydrogen atom, in analog 4 the carbonyl moiety was replaced by hydrogen atoms, and in analogs 5 and 6, the phenyl ring was replaced by cyclohexane moieties. (C) Glide score of naringenin and its structural analogs.
